# A computational-based update on microRNAs and their targets in barley (*Hordeum vulgare *L.)

**DOI:** 10.1186/1471-2164-11-595

**Published:** 2010-10-22

**Authors:** Moreno Colaiacovo, Annalisa Subacchi, Paolo Bagnaresi, Antonella Lamontanara, Luigi Cattivelli, Primetta Faccioli

**Affiliations:** 1 CRA-Genomics Research Centre, via S.Protaso 302, I-29107 Fiorenzuola d'Arda (Pc), Italy

## Abstract

**Background:**

Many plant species have been investigated in the last years for the identification and characterization of the corresponding miRNAs, nevertheless extensive studies are not yet available on barley (at the time of this writing). To extend and to update information on miRNAs and their targets in barley and to identify candidate polymorphisms at miRNA target sites, the features of previously known plant miRNAs have been used to systematically search for barley miRNA homologues and targets in the publicly available ESTs database. Matching sequences have then been related to Unigene clusters on which most of this study was based.

**Results:**

One hundred-fifty-six microRNA mature sequences belonging to 50 miRNA families have been found to significantly match at least one EST sequence in barley. As expected on the basis of phylogenetic relations, miRNAs putatively orthologous to those of *Triticum *are significantly over-represented inside the set of identified barley microRNA mature sequences.  Many previously known and several putatively new miRNA/target pairs have been identified. When the predicted microRNA targets were grouped into functional categories, biological processes previously known to be regulated by miRNAs, such as development and response to biotic and abiotic stress, have been highlighted and most of the target molecular functions were related to transcription regulation.  Candidate microRNA coding genes have been reported and genetic variation (SNPs/indels) both in functional regions of putative miRNAs (mature sequence) and at miRNA target sites has been found.

**Conclusions:**

This study has provided an update of the information on barley miRNAs and their targets representing a foundation for future studies.  Many of previously known plant microRNAs have homologues in barley with expected important roles during development, nutrient deprivation, biotic and abiotic stress response and other important physiological processes. Putative polymorphisms at miRNA target sites have been identified and they can represent an interesting source for the identification of functional genetic variability.

## Background

MicroRNAs (miRNAs) are a class of non-coding small RNAs with fundamental roles in key plant biological processes such as development, signal transduction and environmental stress response [1]. miRNAs act on gene regulation at post-transcriptional level, a phenomenon known in plants as PTGS (Post Transcriptional Gene Silencing), through sequence-based interaction with target mRNAs.

Many plant species have been investigated during recent years for miRNAs identification and characterization. The current information available on barley refers to two papers [2,3]. In particular, the paper of Dryanova et al. reports detailed information on both targets and miRNA coding sequences from *Hordeum vulgare *and for other members of *Triticeae tribe*, to which barley belongs [2]. However, extensive studies describing the organization of miRNA families, specifically in barley, are not yet available (at the time of this writing) and no miRNAs have been deposited in the publicly available miRNA database (miRBase, http://www.mirbase.org), this despite the economic importance of barley and its role as model species for *Triticeae *[4].

The conservation of miRNA sequences across species provides a powerful tool for the identification of novel miRNA genes based on homology with miRNAs previously described in other species. Search based on evolutionary conservation has allowed the identification of miRNA families in many plant species, including those where the complete genome sequence is not available, as it is currently the case of barley. Without genome sequence information a powerful alternative data source comes from ESTs (Expressed Sequence Tags): currently 501,616 ESTs are available in barley http://www.ncbi.nlm.nih.gov/dbEST/dbEST_summary.html[5].

The identification of target genes is a fundamental step for the determination of the biological function of microRNAs, besides being an indirect evidence for their existence. Evolutionary conserved targets have proven very helpful to test the effectiveness of miRNA target detection. The perfect or near perfect complementarity between a miRNA and its target mRNA, that is a peculiar feature of plant miRNAs, gives a powerful tool for the identification of target genes through BLAST analysis of miRNA mature sequences *vs *EST/genomic sequences. A large part of the "*in silico*" predicted targets have then been confirmed as *bona fide *targets by experimental approaches including Northern, 5'-RACE and, more recently, degradome analysis via NGS (Next Generation Sequencing) [6,7].

The correct binding of miRNA to its cognate mRNA is critical for regulating the mRNA level and protein expression. This binding can be affected by single-nucleotide polymorphisms or indels in the miRNA target site leading to the suppression of existing binding sites or the generation of illegitimate ones. Therefore, small polymorphisms in miRNA targets can have a relevant effect on gene and protein expression and represent a type of genetic variability that can influence agronomical traits. As an example, overexpression of miR156b and miR156h in rice results in severe dwarfism, strongly reduced panicle size and delayed heading date [8].

To extend and to update information about miRNAs and their targets in barley and to identify candidate polymorphisms at microRNA target sites, barley EST sequences have been screened and related to Unigene clusters. UniGene is an experimental system for partitioning transcript sequences into a non-redundant set of gene-oriented clusters. Thus each UniGene cluster contains sequences that appear to come from the same transcription locus (gene or expressed pseudogene) http://www.ncbi.nlm.nih.gov/UniGene/index.html. Mining SNPs from ESTs allows the exploitation of genetic variability based on published sequences and the analysis of Unigene clusters can be very helpful for this purpose [9].

## Results and Discussion

### Barley miRNAs

Since only mature miRNA sequences rather than precursor sequences are conserved among plant species, mature miRNA sequences have been used as queries for BLAST search against *Hordeum vulgare *ESTs [10]. One hundred-fifty-six microRNA mature sequences belonging to 50 miRNA families have been found to significantly match at least one EST sequence in barley (the total number of matching ESTs was 855 - as reported in additional files 1 and 2) and could actually be related both to target or miRNA sequences, even if the probability is lower for the latter. Indeed the estimated frequency of pri-miRNAs in *T. aestivum *EST collection is as low as 0.003% [2].

The results illustrated above have been compared with those reported by Dryanova et al. where miRNAs and their targets have been searched in the *Triticeae *tribe [2]. Among the 33 miRNA families identified by Dryanova et al. in at least one species of the *Triticeae *tribe, 22 families were found in barley and 17 of them overlap with the present findings. Regarding barley, some miRNA families were found in just one of the two papers. Dryanova et al. found evidences for 5 additional miRNA families while the present work has found evidences in barley for miR390 and miR396 previously reported only in *T. aestivum*, and for additional 31 families not found by Dryanova et al. in anyone of the investigated species (i.e. miR442, miR529). The reasons for these discrepancies can be ascribed to the different miRBase release used (miRBase Release 8.0 for Dryanova et al., 2008 and miRBase Release 13.0 in the present work) and partially to differences in the BLAST settings adopted. Monocot-specific miRNAs (i.e. miR444) have also been found in both works [11].

Statistical analysis was employed to identify over and under-represented plant species from which the corresponding barley miRNA comes from. As reported in table 1 and 2, barley miRNA sequences putative orthologous to those of *Triticum *are significantly over-represented in our data also when very stringent p-value, e.g. 0.001, was used. *Hordeum *and *Triticum *genera are both members of the *Poaceae *family, *Pooideae *subfamily, *Triticeae *tribe. *H. vulgare *is often used as a model species for *Triticeae*, thanks to its diploid genome that could facilitate genome-wide searches of miRNAs.

**Table 1:** Statistical analysis for the identification of over and under-represented plant species.

	Initial dataset		*H.vulgare*		
	
	n° of mature sequences (redundant set)	%	n° of mature sequences (redundant set) matching at least one barley EST	%	p-value
*Arabidopsis thaliana*	207	10.7	43	8.7	0.019

*Oryza sativa*	415	21.5	102	20.5	0.038

*Glycine max*	79	4.1	15	3.0	0.046

*Pinus taeda*	38	2.0	6	1.2	0.068

*Triticum aestivum*	32	1.7	20	4.0	2.0 × 10^-4^

*Physcomitrella patens*	281	14.6	39	7.8	1.7 × 10^-6^

*Populus trichocarpa*	237	12.3	71	14.3	0.021

*Chlamydomonas reinhardtii*	84	4.4	2	0.4	6.3 × 10^-8^

*Selaginella moellendorffii*	64	3.3	12	2.4	0.058

*Vitis vinifera*	140	7.3	47	9.5	0.012

*Brassica napus*	44	2.3	19	3.8	0.010

*Gossypium hirsutum*	13	0.7	4	0.8	0.185

*Medicago truncatula*	46	2.4	8	1.6	0.068

*Solanum lycopersicum*	30	1.6	11	2.2	0.065

*Sorghum bicolor*	72	3.7	27	5.4	0.014

*Zea mays*	98	5.1	48	9.7	1.1 × 10^-5^

*Brassica oleracea*	7	0.4	2	0.4	0.268

*Brassica rapa*	19	1.0	8	1.6	0.061

*Saccharum officinarum*	16	0.8	11	2.2	2.3 × 10^-3^

*Gossypium herbecium*	1	0.1	0	0.0	0.773

*Carica papaya*	1	0.1	0	0.0	0.773

*Vigna unguiculata*	1	0.1	0	0.0	0.773

*Lotus japonicus*	2	0.1	0	0.0	0.597

*Gossypium rammindii*	2	0.1	2	0.4	0.079

Total	1929		497		

For each species, the table shows the number of mature sequences from the redundant set of 1929 sequences stored in the miRBase and number of mature sequences matching at least one barley EST. It also shows the p-value calculated with a binomial distribution.

**Table 2:** Over and under-represented plant species within barley miRNAs identified with respect to the stringency chosen for the p-value.

Threshold	Over-represented plant species	Under-represented plant species
p-value ≤0.05	*Triticum aestivum*	*Arabidopsis thaliana*
	*Populus trichocarpa*	*Oryza sativa*
	*Vitis vinifera*	*Glycine max*
	*Brassica napus*	*Physcomitrella patens*
	*Sorghum bicolor*	*Chlamydomonas reinhardtii*
	*Zea mays*	
	*Saccharum officinarum*	

p-value ≤ 0.01	*Triticum aestivum*	*Physcomitrella patens*
	*Zea mays*	*Chlamydomonas reinhardtii*
	*Saccharum officinarum*	

p-value ≤ 0.005	*Triticum aestivum*	*Physcomitrella patens*
	*Zea mays*	*Chlamydomonas reinhardtii*
	*Saccharum officinarum*	

p-value ≤ 0.001	*Triticum aestivum*	*Physcomitrella patens*
	*Zea mays*	*Chlamydomonas reinhardtii*

*Zea mays *is also closely related to barley being part of monocot group and *Poaceae *family. *Oryza sativa *although is part of *Poaceae *family is under-represented, when a low stringent p-value (0.05) was used.

Some ESTs have matched to more than one miRNAs belonging either to the same family or to different families (additional file 3). The first case can be due to the high level of similarity among mature sequences from different members of the same family, while ESTs matching to different miRNA families could represent examples of multi-microRNA based control.

Transcripts targeted by more than one miRNA have also been found also in other plant species such as rice [12]. These findings are common in animals where many different miRNAs recognize the same target mRNA, usually at the 3'UTR [13].

To identify and annotate potential microRNA-regulated genes in barley, the 855 matching ESTs were related to Unigene clusters. Clusters annotated as protein-coding sequences were then selected for subsequent analysis and listed in tables 3 and 4. A total of 121 different Unigene clusters putatively representing the targets for 37 miRNA families has been found. Similar results (e.g. on average more than 1 putative target/miRNA family) were reported by Zhang et al. in maize (115 target for 26 miRNA families) [14]. Sometimes different targets for a specific miRNA are members of the same gene family (e.g. miR156-SBP family), while in other cases there is no evident relationship among the putative targets of a given miRNA (e.g. miR1121). Previous studies report six targets or fewer for most Arabidopsis miRNAs, a number significantly lower than in animals, for example, in Drosophila each miRNA has on average over 50 predicted targets [13,15].

**Table 3:** miRNA target genes identified in barley and confirmed by previous studies

miRNA family	miRNA name	Unigene	Unigene annotation	Literature reported target for this miRNA (citation number in brackets)
156	miR156	Hv.29207	protein coding (SBP domain)	*Arabidopsis thaliana *[24]
	
	miR156	Hv.5875	protein coding (SBP domain)	*Oryza sativa *[8]
	
	miR156	Hv.28351	protein coding (SBP domain)	*Hordeum vulgare *[2]
	
	miR156	Hv.21387	SPL2 (SQUAMOSA PROMOTER BINDING PROTEIN-LIKE 2)	*Triticum aestivum *[2]
	
	miR156	Hv.28414	SPL5 (SQUAMOSA PROMOTER BINDING PROTEIN-LIKE 5)	

				*Arabidopsis thaliana *[24]
159	miR159	Hv.12	MYB family transcription factor	*Oryza sativa *[39]
				*Hordeum vulgare *[2]
				*Triticum aestivum *[2]

				*Arabidopsis thaliana *[24]
160	miR160	Hv.5089	ARF16 (AUXIN RESPONSE FACTOR 16)	*Oryza sativa *[39]
				*Hordeum vulgare *[2]
				*Triticum aestivum *[2]

164	miR164	Hv.877	NAC domain containing protein	*Arabidopsis thaliana *[24]
	
	miR164	Hv.28795	NAC domain containing protein	*Zea mays *[14]
	
	miR164	Hv.25370	NAM superfamily	*Hordeum vulgare *[2]
	
	miR164	Hv.21779	NAC domain containing protein	*Triticum aestivum *[2]

168	miR168	Hv.26206	AGO1 (ARGONAUTE 1)	*Arabidopsis thaliana *[24]
	
	miR168	Hv.19452	AGO1 (ARGONAUTE 1)	*Hordeum vulgare *[2]
				*Triticum aestivum *[2]

169	miR169	Hv.13681	CCAAT-binding transcription factor (CBF-B/NF-YA) family protein	*Aquilegia coerulea *[40]
	
	miR169	Hv.406	CCAAT-binding transcription factor (CBF-B/NF-YA) family protein	*Hordeum vulgare *[2]
	
	miR169	Hv.9532	CCAAT-binding transcription factor (CBF-B/NF-YA) family protein	*Triticum aestivum *[2]

				*Arabidopsis thaliana *[24]
171	miR171	Hv.9855	GRAS family transcription factor	*Brachypodium distachyon *[41]
				*Hordeum vulgare *[2]
				*Triticum aestivum *[2]

				*Arabidopsis *[42]
172	miR172	Hv.6575	RAP2.7/TOE1 (TARGET OF EAT1 1), AP2 superfamily	*Hordeum vulgare *[2]
				*Triticum aestivum *[2]

393	miR393	Hv.29376	AFB2 (AUXIN SIGNALING F-BOX 2), auxin binding/ubiquitin-protein ligase	*Aquilegia coerulea *[40]

	miR393	Hv.2498	TIR1 (TRANSPORT INHIBITOR RESPONSE 1), ubiquitin-protein ligase	*Hordeum vulgare *[2]
				*Triticum aestivum *[2]

				*Aquilegia coerulea *[40]
394	miR394	Hv.8877	F-box family protein	*Hordeum vulgare *[2]
				*Triticum aestivum *[2]

				*Aquilegia coerulea *[40]
395	miR395	Hv.12870	ATPS1	*Hordeum vulgare *[2]
				*Triticum aestivum *[2]

396	miR396	Hv.28722	WRC, QLQ	
	
	miR396	Hv.22031	growth-regulating factor	*Arabidopsis thaliana *[39]
	
	miR396	Hv.19321	WRC, QLQ	*Oryza sativa *[39]
	
	miR396	Hv.9742	WRC, QLQ	*Triticum aestivum *[2]

399	miR399	Hv.5443	ATUBC24/PHO2/UBC24 (PHOSPHATE 2), ubiquitin-protein ligase	*Arabidopsis thaliana *[39]
				*Oryza sativa *[39]

408	miR408	Hv.10831	ARPN (PLANTACYANIN), copper ion binding (Cu-bind-like superfamily)	*Medicago truncatula *[43]
	
				*Populus trichocarpa *[44]
	miR408	Hv.24052	Plastocyanin-like domain-containing protein (Cu-bind-like superfamily)	*Oryza sativa *[45]
				*Hordeum vulgare *[2]
				*Triticum aestivum *[2]

	miR408	Hv.20945	ARGONAUTE like superfamily	*Hordeum vulgare *[2]

529	miR529	Hv.29207	protein coding (SBP domain)	*Aquilegia coerulea *[40]
	
	miR529	Hv.28351	protein coding (SBP domain)	*Zea Mays *[46]

				*Arabidopsis thaliana *[47]
827	miR827	HV. 10218	SPX superfamily, MFS superfamily	*Oryza sativa *[48]

**Table 4:** Novel miRNA target genes identified

miRNA family	miRNA name	Unigene	Unigene annotation	Functional annotation
390	miR390	Hv.15993	protease inhibitor, seed storage, lipid transfer protein (LTP) family protein	lipid transport

441	miR1126	Hv.10635	beta-adaptin	protein transport

	miR1126	Hv.25101	ankyrin protein kinase, serine/threonine protein kinase	regulation in signal transduction

	miR1126	Hv.18172	protein coding	unknown function

	miR1126	Hv.5267	SRT2, DNA binding	vernalization, auxin signalling

818	miR818+1436	Hv.11323	protein coding	unknown function

	miR818+1436	Hv.9623	NLI interacting factor (NIF) family protein	phosphatase activity

	miR1436	Hv.8609	Coproporphyrinogen III oxidase	chlorophyll biosynthesis

	miR1436	Hv.16854	P-loop NTPase superfamily	unknown function

	miR1436	Hv.8351	protein coding	unknown function

	miR1436	Hv.28025	protein coding	unknown function

	miR1436	Hv.27779	Vps51 superfamily	vescicular transport

	miR1436	Hv.19811	ILL3 (IAA-amino acid hydrolase ILR1-like 3), metallopeptidase	stress and hormone response

	miR1436	Hv.18734	MAP kinase	signal transduction, stress signalling

	miR1436	Hv.15543	protein coding	unknown function

	miR1436	Hv.12920	PKc-like superfamily	abiotic stress resistance

	miR1436	Hv.11057	Integral membrane family protein	endomembrane system

	miR1436	Hv.3476	protein coding	unknown function

	miR1439	Hv.19109	PKc-like superfamily	unknown function

	miR1439	Hv.23816	exo-endo-phos superfamily	unknown function

	miR1439	Hv.11224	tatD-related deoxyribonuclease family protein	deoxyribonuclease activity

821	miR821	Hv.3660	GDH1 (Glutamate dehydrogenase)	nitrogen metabolism

1030	miR1030	Hv.12064	AS1/ATMYB91/ATPHAN/MYB91 (ASYMMETRIC LEAVES 1, MYB DOMAIN PROTEIN)	transcription factor

	miR1030	Hv.7960	protein coding	unknown function

	miR1030	Hv.14867	RNA recognition motif (RRM)-containing protein	post-transcriptional gene expression processes

1119	miR1119	Hv.29225	protein coding	unknown function

	miR1119	Hv.29210	protein coding	unknown function

	miR1119	Hv.27666	protein coding	unknown function

	miR1119	Hv.23883	ADF2 (ACTIN DEPOLYMERIZING FACTOR 2), actin binding	actin turnover, stress response, plant defense signalling pathway

	miR1119	Hv.23689	RRM superfamily, RNA binding	involved in post-transcriptional gene expression processes

	miR1119	Hv.23343	molybdenum cofactor sulfurase family protein, superfamily	stress response

1120	miR1120	Hv.21827	protein coding	unknown function

	miR1121	Hv.464	serine/threonine kinase	response to salt stress

	miR1121	Hv.20180	Kelch repeat-containing protein	unknown function

	miR1121	Hv.2132	protein coding	unknown function

	miR1121	Hv.26959	POK (POKY POLLEN TUBE)	pollen tube growth

	miR1121	Hv.20763	SRG1 (SENESCENCE-RELATED GENE 1), oxidoreductase	flavonoid biosyntetic processes and senescence

	miR1121	Hv.20600	serine/threonine protein kinase, PKc-like superfamily	abiotic stress resistance

	miR1121	Hv.12124	ATPase family AAA domain-containing protein	unknown function
	miR1121	Hv.10391	protein coding	unknown function

	miR1121	Hv.9294	protein coding	unknown function

	miR1121	Hv.6581	protein coding	unknown function

	miR1121	Hv.6532	ATPase-Plipid, haloacid dehalogenase-like hydrolase family protein	ATPase activity

	miR1121	Hv.4756	FAR1 superfamily, MULE transposon domain	light control of development

	miR1121	Hv.3142	CRS1-YhbY (CRM domain) superfamily	RNA binding/intron splicing

1122	miR1122	Hv.12219	serine/threonine protein kinase, PKc-like superfamily	abiotic stress resistance

	miR1128+1133	Hv.23560	indole-3-glycerol phosphate synthase, TIM-phosphate binding superfamily	aminoacid biosynthesis

	miR1128+1133	Hv.679	UBIQUITIN CARRIER PROTEIN, ubiquitin-protein ligase	ubiquitination

	miR1128+ 1133+1136	Hv.26146	AIM1 (ABNORMAL INFLORESCENCE MERISTEM), enoyl-CoA hydratase	auxin metabolism

	miR1128+1135	Hv.23257	integral membrane HPP family protein	unknown function

	miR1128+1135	Hv.21122	SOS5 (SALT OVERLY SENSITIVE 5)	salt signalling/osmo-stress

	miR1128	Hv.17314	protein coding	unknown function

	miR1128	Hv.14876	ARF-GAP DOMAIN, C2 superfamily	vescicle traffic/development

	miR1128+1133	Hv.12752	ATP-dependent peptidase, ATPase, metallopeptidase	peptidase activity

	miR1128+ 1133+1136	Hv.6454	oligopeptide transporter	oligopeptide transporter

	miR1128+1133	Hv.3596	Cysteine hydrolases, catalytic/nicotinamidase	response to abscisic acid stimulus

	miR1133	Hv.14592	pathogenesis related protein-1	plant defense

	miR1133	Hv.12091	oxidoreductase, zinc-binding dehydrogenase family protein	stress response

	miR1133	Hv.28954	HLH superfamily	transcription factor

	miR1133	Hv.28555	serine/threonine protein kinase, PKc-like superfamily	abiotic stress resistance

	miR1133	Hv.4244	CTP synthase	CTP synthase activity

	miR1135	Hv.5272	Epidermal growth factor receptor-like protein	vacuolar transport

	miR1135	Hv.223	Limit dextrinase	carbohydrate metabolic process

	miR1135	Hv.18515	ubiquitin family protein	ubiquitination

	miR1135	Hv.16976	HEAT repeat-containing protein	unknown function

	miR1135	Hv.16897	ATTPS6 (A. thaliana trehalose phosphatase/synthase 6), transferase, transferring glycosyl groups, trehalose-phosphatase	development

1130	miR1130	Hv.12920	PKc-like superfamily	abiotic stress response

1134	miR1134	Hv.29810	WRKY transcription factor	transcription factor

	miR1134	Hv.29222	ribulose-1,5-bisphosphate carboxylase/oxygenase large subunit	carbon fixation

	miR1134	Hv.22973	octopine synthase binding factor1, ATBZIP53 (BASIC REGION/LEUCINE ZIPPER MOTIF 53), DNA binding/protein heterodimerization/sequence-specific DNA binding/transcription factor	stress response

	miR1134	Hv.22600	fumarylacetoacetate hydrolase family protein	tyrosine catabolism

	miR1134	Hv.9579	L-asparaginase, putative/L-asparagine amidohydrolase, putative	nitrogen metabolism

	miR1134	Hv.239	AWPM-19-like membrane family protein	freezing tolerance

	miR1134	Hv.26138	AWPM-19-like membrane family protein	freezing tolerance

	miR1134	Hv.24001	dehydrin family protein	stress response

	miR1134	Hv.23108	B3-hordein fragment	seed storage protein

	miR1134	Hv.23080	ATNUDT17 (A. thaliana Nudix hydrolase homolog 17)	hydrolase activity

	miR1134	Hv.16060	Sulfotransferase domain	sulfotransferase activity

1438	miR1438	Hv.26216	RAP2.2, AP2 superfamily	transcription factor

1533	miR1533	Hv.29041	aldehyde dehydrogenase	stress response

1846	miR1846	Hv.19467	UDP-GLUCOSYL TRANSFERASE	stress response

1848	miR1848	Hv.6944	Pollen_Ole_e_I super family	unknown function

1862	miR1862	Hv.26602	protein coding	unknown function

1867	miR1867	Hv.18578	FLAVODOXIN-LIKE QUINONE REDUCTASE 1	auxin response gene

	miR1867	Hv.1368	ATPase, coupled to transmembrane movement of substances	ATPase activity

1871	miR1871	Hv.28885	protein coding	unknown function

2091	miR2091	Hv.6058	FKBP superfamily	regulation of photosyntetic process/stress response/plant hormone pathways

2094	miR2094	Hv.699	RNA binding	RNA binding

2102	miR2102	Hv.22799	RNA binding	stress response

Although several of the candidate miRNA/target pairs here identified have the same functional annotation reported in previously studied species (table 3) and specifically in barley some putative novel microRNA/target pairs have been discovered (table 4) [2]. Actually, some of these novel targets were reported by literature as regulated by a different microRNA. Most of the novel miRNA/target pairs refer to miRNAs recently discovered and thus probably less studied (i.e. miR1120, miR1122, miR1134). The Argonaute-like protein found as a novel target for miR408 in *H.vulgare *by Dryanova et al. has been confirmed also in the present work.

Transcription factor families comprise most of the highly conserved miRNA targets (see table 3) such as SBP family for miRNA 156, AP2 family for miR172, GRAS family for miR171, myb family for miR159, GRF family for miR396 and ARF family for miR160. These results confirmed what previously observed in *Triticeae *and in other species [2]. In rice about 70% of conserved miRNA targets are transcription factors, while in wheat one-third of the predicted targets was found to encode for transcription factors [11,12]. Conserved miRNAs also target genes involved in their own biogenesis and function: as an example miR168 targets AGO1 which is part of the RISC complex responsible for the miRNA-mediated mRNA cleavage [15]. miRNA regulate gene expression also by targeting enzymes of the ubiquitination pathway (ubiquitin conjugating enzyme E2 and TIR1/ubiquitin ligase): barley miR393, miR399, miR1128, miR1133, miR1135 can be considered putative regulators of gene expression at protein level.

The number of target genes identified as different Unigene clusters (tables 3-4) is very different among the miRNA families. In rice Zhou et al. have found a high number of targets for miR156 and miR396 and a low number for miR162, miR167, miR395, miR398 and miR399 [12]. This finding could indicate that the former miRNAs are nodes in gene regulation networks, while the latter could act on specialized pathways.

**Figure 1:**
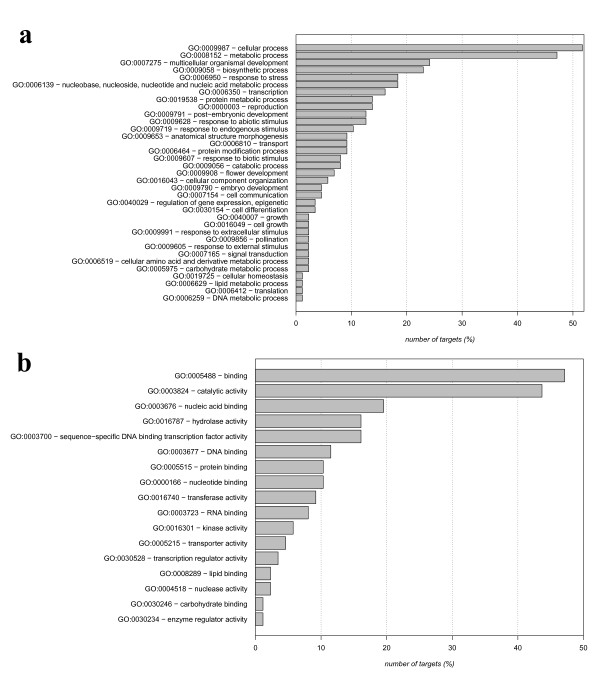
**Functional enrichment for the miRNA targets identified**. For each GO term it is shown the number of targets annotated with that term with respect to the total number of targets (%). Figure 1a refers to the biological process, while figure 1b refers to the molecular function.

**Figure 2:**
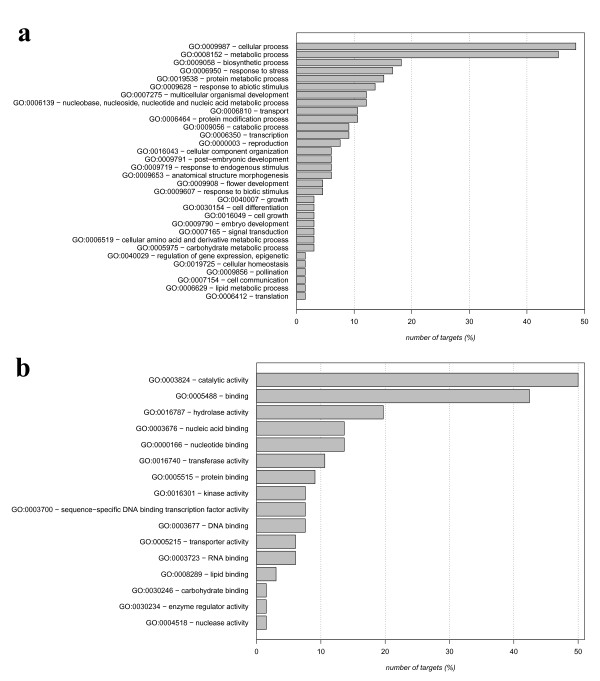
**Functional enrichment for the novel miRNA targets identified**. For each GO term it is shown the number of targets annotated with that term with respect to the total number of novel targets (%). Figure 2a refers to the biological process, while figure 2b refers to the molecular function.

The predicted targets have been grouped into functional categories and reported in figures 1 and 2 where the target annotations based on GO terms are shown. Biological processes known to be regulated by miRNAs, such as development and response to biotic and abiotic stress, have been highlighted both in known (figure 1a) and in novel targets (figure 2a). Moreover, most of the molecular functions are related to transcriptional regulation and DNA/nucleotide binding in both groups (figures 1b-2b). These findings suggest that the predicted target genes can be considered a reliable dataset to be used in subsequent analysis.

For some Unigene clusters the annotation was related to transcribed genes rather than protein coding sequences. These Unigenes could represent miRNA-coding genes as shown by other authors [16,17]. Table 5 reports the Unigene clusters candidate to encode miRNA coding genes on the basis of the precursor sequence secondary structure (MFEI >0.85, see Materials and Methods) and of the presence of the miRNA* (miRNA passenger sequence). It cannot be excluded that the clusters unable to fold with a miRNA-like structure (e.g. Hv.8579, Hv.11623) are false negatives for several reasons, such as truncated precursor sequences in EST database. Putative microRNA sequences have also been BLASTed against previously known precursors available from mirBASE: the analysis found similarities with 6 different miRNA families. The secondary structures of the putative microRNA precursors are reported in the additional file 4. Linking together sequences containing miRNA precursors from Dryanova et al. and from the present work, information on several microRNA putative secondary structures, belonging to 10 miRNA families are now available [2]. The mature miRNAs predicted from these data are 18 to 24 nt long, with a higher frequency for 20 and 21 nt.

**Table 5:** Unigene clusters candidate to encode for miRNAs

	Features of the precursors identified						Best scoring alignment with miRBase precursors			
Unigene cluster	ΔG (kcal/mol)	MFEI	NM	ML	PL	Arm	Accession number	miRNA	Score	e-value

Hv.1306	-72.8	0.89	2	21	167	3'	MI0006178	tae-MIR444	736	1 × 10^-54^

Hv.5064	-22.0	1.00	2	18	68	3'	MI0006199	tae-MIR1137	168	4 × 10^-8^

Hv.7117	-74.9	0.96	3	21	115	5'	MI0011565	bdi-MIR397	196	5 × 10^-10^

Hv.8158	-60.7	0.88	3	21	92	5'	MI0001763	sof-MIR168a	393	1 × 10^-26^

Hv.14657	-31.5	1.85	2	21	69	3'	MI0006183	tae-MIR1121	241	3 × 10^-14^

Hv.15131	-51.1	0.90	2	21	129	3'	MI0006976	osa-MIR444d	431	2 × 10^-29^

Hv.16635	-91.7	0.92	3	21	200	3'	MI0006170	tae-MIR159a	805	2 × 10^-60^

Hv.22601	-34.0	0.92	4	22	97	3'	MI0006192	tae-MIR1130	179	9 × 10^-9^

Hv.28058	-63.8	1.60	2	24	129	3'	MI0006182	tae-MIR1120	147	7 × 10^-6^

Hv.29065	-53.6	1.07	4	22	131	5'	MI0006199	tae-MIR1137	182	8 × 10^-9^

Hv.29519	-42.9	1.02	2	21	96	3'	MI0006192	tae-MIR1130	144	8 × 10^-6^

Hv.30469	-39.0	0.91	3	21	117	5'	MI0006199	tae-MIR1137	141	2 × 10^-5^

For each cluster, the table shows details about the putative precursors: the free energy ΔG, the minimal folding free energy index (MFEI), the number of mismatches in miRNA/miRNA* duplex (NM), the mature length (ML), the precursor length (PL) and the location of mature miRNA (3' or 5'). Moreover, it is also reported the more similar known precursor in miRBase, with the alignment score and p-value.

### Genetic variation at miRNA target sites

A single nucleotide change in the sequence of a target site can affect miRNA regulation: as a consequence naturally occurring SNPs in target sites are candidates for relevant functional variations. Nair et al. established a perfect association between a SNP at the miR172 targeting site and cleistogamy in barley [18]. Overall few papers have been published to date describing variations among plant genotypes at miRNAs and their target sites, while plenty of information is available for humans [19-23]. Genome-wide studies in humans have shown that the levels of polymorphism at miRNA and miRNA target sites are lower than at coding or neutral regions, however beneficial miRNA-target site polymorphisms also exist [19].

In this study, publicly available SNP data have been analyzed in context with miRNAs and their target sites. EST-derived SNPs can provide a rich source of biologically useful genetic variation due to the redundancy of gene sequence, the diversity of genotypes present in the databases and the fact that each putative polymorphism is associated with an expressed gene. Variations both in functional regions of putative miRNAs (mature sequence) and at miRNA target sites have been found. Previous works in human have highlighted a relatively low level of variation in functional microRNA regions and an appreciable level of variation at target sites [21].

**Figure 3:**
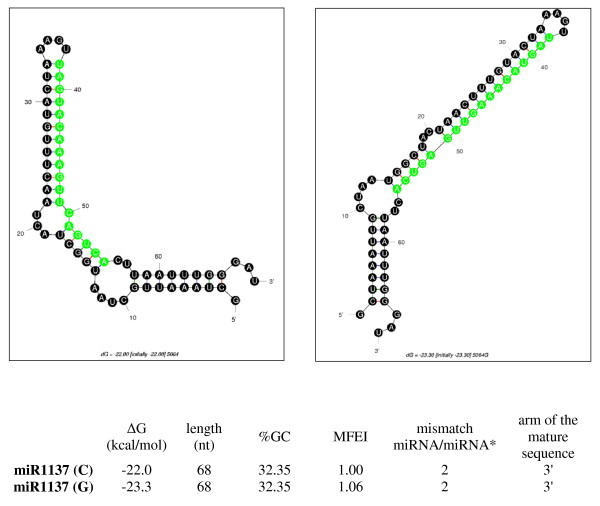
**Predicted secondary structures of the two variants of the miR1137 precursor identified in the Unigene cluster Hv.5064**. The variants with a C and a G in the 13^th ^position are respectively reported in the left and in the right side of the figure. The table shows for each variant: the free energy ΔG, the length of the precursor, the GC content, the MFEI (Minimal Folding Energy Index), the number of mismatches between the mature sequence and the paired miRNA* passenger and the arm of the hairpin where the mature sequence is located.

Hv.5064, the candidate for miR1137 coding sequence, has been tested for modifications of pre-miRNA structure due to a base substitution in position 13 (**C/G**, table 6, figure 3). To evaluate the possible impact of this SNP on pre-miRNA secondary structure, Gibbs free energy (ΔG) and MFEI from each version of pre-miRNA were calculated using mfold program. Data in figure 3 show the structural variation obtained when moving from "C variant" to "G variant" with a higher MFEI for the second one and thus a greater stability of the molecule (miRNA-miRNA* pairing enhanced in the G variant). Difference in ΔG moving from C to G and vice versa were calculated according to Ehrenreich and Purugganan [19]. ΔΔG was +1.3 for the former change and -1.3 for the latter suggesting that some SNPs can stabilize/destabilize pre-miRNA structure. No target gene has been reported in literature for miR1137.

**Table 6:** Putative polymorphisms identified at miRNA target sites and inside miRNA mature sequences

miRNA family	miRNA name	Unigene	Unigene cluster annotation	Putative Polymorphisms at miRNA target site (5'-3')	Barley miRNA mature sequence (5'-3')
156	miR156	Hv.5875	protein coding (SBP domain)	#GTGCTCTC**T**(**C**)CTCTTCTGTCA	UGACAGAAGAG**A**GAGAGCAC (12)

	miR156	Hv.21387	SPL2 (SQUAMOSA PROMOTER BINDING PROTEIN-LIKE 2)	#ATGCTCT**C**(G)T**C**(T)T**C**(G)TTCTGTCA	UGACAGAA**G**A**G**A**G**AGAGCAU (9-11-13)

164	miR164	Hv.28795	NAC domain containing protein	#AGCAAGTGCC**C**(A)TGCTTCTCCA	UGGAGAAGCA**G**GGCACUUGCU (11)

169	miR169	Hv.13681	CCAAT-binding transcription factor (CBF-B/NF-YA) family protein	#CAGGCAACTCATCCTTGGC**T**(C)T	A**A**GCCAAGGAUGAGUUGCCUG (2)

	miR169	Hv.9532	CCAAT-binding transcription factor (CBF-B/NF-YA) family protein	#GGCAATTCATCCTTGG**C**(T)TT	AA**G**CCAAGGAUGAAUUGCC (3)

393	miR393	Hv.2498	TIR1 (TRANSPORT INHIBITOR RESPONSE 1), ubiquitin-protein ligase	#**G**(C)ACAATGC**G**(T)ATCCC(+CT)TTTGGA	UCCAAA**()**GGGAU**C**GCAUUGU**C **(6-12-20)

396	miR396	Hv.9742	WRC, QLQ	#GTTCAA**G**(A)AAAGCCTGTGGA	UCCACAGGCUUU**C**UUGAAC(13)

408	miR408	Hv.20945	ARGONAUTE like superfamily	#CAGGGCA**G**(T)AGGCAGTGCAG	CUGCACUGCCU**C**UGCCCUG (12)

	miR408	AutoSNP contig 2094	Plastocyanin	#CAGGGAAGAGGC**A(C)**GTGCGG	CCGCAC**U(G)**GCCUCUUCCCUG (7)

444	miR444	Hv.16297	/		*GCAGUUGC**U(C)**GCCUCAAGCUU (9)

818	miR818	Hv.11323	protein coding	#CCGTCCCATA**A**(CC)TATAAGGG	CCCUUAUA**U**UAUGGGACGG (9)

	miR1436	Hv.8351	protein coding	#ACTCCCTC**C(T)**GTCCCATAAT	AUUAUGGGAC**G**GAGGGAGU (11)

	miR1436	Hv.11323	protein coding	#ACTCCCTCCGTCCCATAA**--**(CC)T	A**--**UUAUGGGACGGAGGGAGU (2)

	miR1439	Hv.23816	exo-endo-phos superfamily	# AATACTCACTCCGTCCCAAA**A**(G)	**U**UUUGGGACGGAGUGAGUAUU (1)

	miR1439	Hv.11224	tatD-related deoxyribonuclease family protein	#TACTCACTCCGTTCC**A(T)**AAA	UUU**U**GGAACGGAGUGAGUA (4)

821	miR821	Hv.3660	GDH1 (Glutamate dehydrogenase)	#TCA**A**(C)CAAAAAAGTTGAAT	AUUCAACUUUUUUG**U**UGA (15)

1030	miR1030	Hv.14867	RNA recognition motif (RRM)-containing protein	#TGG**T**(G)GCAGGTGCAGGTGCAGG	CCUGCACCUGCACCUGC**A**CCA (18)

1119	miR1119	Hv.29226	/		*UGG**C**(-)**A**(C)CGGCGCGAUGCUCAGUC**A**(-)**G**(C) (4-5-23-24)

	miR1119	Hv.29225	protein coding	#CTGA**C**(A)TGAGCATCGCGCCGTGCCA	UGGCACGGCGCGAUGCUCA**G**UCAG (20)

	miR1119	Hv.27666	protein coding	#**C**(T)TGA**C**(**T**/A)T**G**(A)**A**(G)GC**A**(T)TCGCGCCGTGCC	GGCACGGCGCGA**U**GC**UC**A**G**UCA**G **(13-16-17-19-23)

	miR1119	Hv.23343	molybdenum cofactor sulfurase family protein, superfamily	#C**T(G)G(T)A(G)**C**T(C)**GAGCATCGCGCCGTGCC	GGCACGGCGCGAUGCUC**A**G**UCA**G (18-20-21-22)

	miR1119	Hv.29210	protein coding	#**T**(G)GGCAC**G**(A)G**C**(T)GCGA**T**(A)GCTCA**G**(A)TCA**G**(A)	**C**UGA**C**UGAGC**A**UCGC**G**C**C**GUGCC**A **(1-5-11-16-18-24)

1120	miR1121	Hv.464	serine/threonine kinase	#**A**(G)**A**(G)GAGCGTTTAGATCACTA	UAGUGAUCUAAACGCUC**UU **(18-19)

	miR1121	Hv.6581	protein coding	#TAAGAGCGTTTAGATCAC**T(C)**A	U**A**GUGAUCUAAACGCUCUUA (2)

	miR1121	Hv.6532	ATPase-Plipid, haloacid dehalogenase-like hydrolase family protein	#TAA**G**(A)AGTGTTTAGATCACTACT	AGUAGUGAUCUAAACACU**C**UUA (19)

	miR1121	Hv.5064	/		*UAGUACAAAGUU**G**(**C**)AGUCA (13)

1122	miR1128	Hv.14876	ARF-GAP DOMAIN, C2 superfamily	#TTT**G**(T)G**G**(A)ACGG**A**(G)GGGAGTAGTA	UACUACUCCC**U**CCGU**C**C**C**AAA (11-16-18)

	miR1133	Hv.12091	oxidoreductase, zinc-binding dehydrogenase family protein	#TTTGG**G**(A)ACGGAGGGAGTA**C**(-)TAT	AUA**G**UACUCCCUCCGU**C**CCAAA (3-16)

	miR1133	Hv.28555	serine/threonine protein kinase, PKc-like superfamily	#TTTCGGACAGAGG**G**(T)AGTATAT	AUAUACU**C**CCUCUGUCCGAAA (8)

	miR1135	Hv.18515	ubiquitin family protein	#TT**C**(G)GGAATTACTTGTCGCA	UGCGACAAGUAAUUCC**G**AA (17)

1134	miR1134	Hv.22973	octopine synthase binding factor1, ATBZIP53 (BASIC REGION/LEUCINE ZIPPER MOTIF 53), DNA binding/protein heterodimerization/sequence-specific DNA binding/transcription factor	#TCTTCTTCTTCTTCTTG**(C)TTC(---)**TTG	CAA**GAAC**AAGAAGAAGAAGAAGA (4-5-6-7)

	miR1134	Hv.9579	L-asparaginase, putative/L-asparagine amidohydrolase, putative	#T**C(G)T(C)**TCTTCTTCTTGGTGTTGGTG	CACCAACACCAAGAAGAAGA**AG**A (21-22)

	miR1134	Hv.26138	AWPM-19-like membrane family protein	#TCTTCTT**C**(G)TTCT**T**(A)GTCGTTGTTG	CAACAACGAC**A**AGAA**G**AAGAAGA (11-16)

	miR1134	Hv.24001	dehydrin family protein	#TTCTTCTTCTTGTTGTTTT**T(-)**G	C**A**AAAACAACAAGAAGAAGAA (2)

	miR1134	Hv.8025	/		*UCUUCUUCUUUUGUUGUUG**U**(C)UG (20)

	miR1134	Hv.5763	/		*CUU**C(G)**UUCCUCUUGUUGUUGUUG (4)

1871	miR1871	Hv.28885	protein coding	# C**(T)**AACATGATATCAGAGCCA	UGGCUCUGAUAUCAUGUU**G **(19)

Letters in bold refer to SNPs represented by at least two independent sequences, while the numbers in brackets refer to the position of the SNP in the sequence. Plus means insertion, minus means deletion.

**Figure 4:**
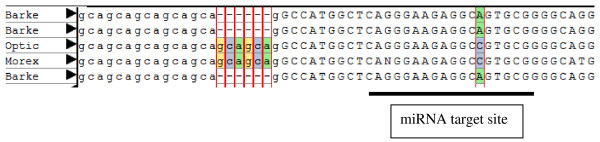
**SNP identified in contig2094 within the target site for miRNA 408**. In this multiple alignment performed with AutoSNP, two cultivars (Optic and Morex) report the same allelic variant as part of a haplotype including a SSR polymorphism located upstream the target sequence.

In plants most of the miRNA-based regulation relies on the cleavage of target mRNAs that normally occurs at the tenth nucleotide of the complementary region and numerous studies on miRNA-target interaction have highlighted the importance of positions 2 to 12, more frequently 10 and 11 [24]. Although most of the putative polymorphisms highlighted in this work are outside those critical positions, several examples of putative functionally relevant polymorphisms have been detected. Table 6 reports the putative polymorphisms detected after comparison among EST sequences inside Unigene clusters, without any selection against false positives. Some of these nucleotide variation could be due to sequencing errors or related to very similar genes belonging to a specific family, nevertheless when the SNPs/indels rely on two or more copies of independent sequences it can be considered a good candidate for a true positive polymorphic target site [25]. For example, a polymorphism in miRNA 408 target site detected by AutoSNP in contig 2094 (coding for a plastocyanin) is based on sequences from two different cultivars reporting the same allelic variant as part of a haplotype where a SSR (Simple Sequence Repeat) polymorphism is located upstream the target sequence (figure 4). Some polymorphisms also showed an evolutionary conserved position, the nucleotide variation identified in Hv.2498 (targeted by miR393) has also been found in the orthologous gene of Arabidopsis in the same position by Ehrenreich and Purugganan [19].

The Squamosa-promoter Binding Protein (SBP) is a known target family for miR156. Many plant transcription factors involved in the regulation of the transition from the vegetative to the reproductive phase belong to this family and it has been shown that overexpressing SBP genes can lead to increased leaf initiation, decreased apical dominance and delayed flowering time [15]. The increase of the activity of some miRNAs (among which miR156) is part of the infection strategy performed by the Turnip mosaic virus in Arabidopsis [26,27]. miR156 performs a critical function in mediating developmental processes and it is also related to the response to biotic stress. The screening of barley databases has identified two SBP genes targeted by miR156 for which two nucleotide variations occur in critical positions (11-12). If these SNPs will be experimentally confirmed, they could have the effect of destabilizing the interaction between the miRNA and the mRNA, which could consequently avoids cleavage and lead to phenotypical variations in developmental features or in the resistance to viral infection.

A SNP also occurs in a crucial point of the experimentally confirmed NAC1 target for miR164. NAC1 is a transcription factor involved in shoot apical meristem formation and auxin-mediated lateral root formation. Guo et al. showed that the overexpression of miR164 leads to reduced lateral rooting; conversely the disruption of the regulation mediated by this miRNA increases the number of lateral roots [28]. The authors have reported that miR164 directs cleavage *in vivo *at a position complementary to the 10^th ^nucleotide from the 5' end of the mature sequence [28]. The SNP found in barley is in the 11^th ^position, therefore it is likely to prevent the cleavage and produce phenotypic effects on root development.

SNPs have been identified also in other two conserved miRNA targets, TIR1 and AGO4, targeted respectively by miR393 and miR408. TIR1 is an auxin-receptor negatively regulated by miRNAs in response to bacterial flagellin, as a defence mechanism against *Pseudomonas syringae *[29]. AGO4 is a protein involved in the siRNA mediated gene silencing, and it is required for the resistance to the same pathogen [30]. Therefore, miR393 and miR408 are likely to work in a coupled manner during *P. syringae *infection. The two SNPs identified are in the 12^th ^position and could potentially alter the levels of pathogens resistance.

SNPs were also found in previously not reported miRNA targets, such as the AWPM-19-like protein matching to the miRNA 1134. AWPM-19 accumulates in wheat plasma membrane during cold acclimation in response to abscisic acid [31]. If this miRNA really controls the synthesis of this protein, a deleterious SNP in the 11^th ^position could then change resistance to cold stress.

## Conclusions

This study has thus provided an update of the information on barley miRNAs and their targets representing a foundation for future studies.

Novel putative target genes have been identified and most of them are involved in stress and hormone response. Indeed, the role of plant miRNAs in abiotic and biotic stress response as well as in auxin signalling is well known [32,33]. In particular, protein kinases such as protein kinase C and serine/threonine kinase, known to be important regulator on abiotic stress resistance, are largely present in novel microRNA/target pairs identified.

The results have also shown that microRNA target sites can be an interesting source for the identification of functional genetic variability, representing an interesting source of candidate molecular markers for application in barley breeding. Putative polymorphisms have now to be verified by amplification and sequencing of the target sequences on a larger set of genotypes.

Sequence analysis based on known miRNAs can obviously give insights only on conserved mRNAs and related targets. Future work will thus be based on the construction of a degradome library for parallel analysis of RNA end (PARE), a powerful approach for high-throughput identification/validation of conserved and non conserved targets.

## Methods

### miRNA reference dataset

The initial miRNA dataset has been obtained by extracting the mature sequences (1929 entries) of the *Viridiplantae *group from the miRBase release 13 http://www.mirbase.org[34]. By removing identical mature sequences, the size of this dataset has been subsequently reduced to 1014 non-redundant sequences related to 468 miRNA families.

### Searching for mature miRNAs matching sequences in barley

The full collection of non-redundant mature miRNA sequences was used in a BLASTn search against dbEST http://www.ncbi.nlm.nih.gov, accepting a number of mismatch lower than 4.

The set of miRNA mature sequences (including the identical sequences removed at the first step of the work) with at least one matching EST have been classified on the basis of the species of origin. The binomial distribution was used to assess the statistical significance for the represented plant species; this allowed identifying those species chosen from the initial dataset more or less frequently than random. Four different thresholds for the p-values were applied (0.05, 0.01, 0.005, 0.001).

Matching ESTs have then been related to Unigene clusters and the corresponding annotations were recorded (if available). The GO slimmer tool available on the Gene Ontology website http://www.geneontology.org has been used to identify the GO slim terms more represented in the set of potential targets on the basis of the Unigene cluster annotations. For this analysis the Plant GO Slim subset has been used.

### Identification of putative miRNA precursors

True miRNA precursors should have both a mature sequence on one arm of the hairpin and a paired passenger sequence (called miRNA*) on the opposite arm. To assess these features the precursor sequences were extracted from the consensus sequences, obtained by the Sequencer Software (Gene Codes) on Unigene cluster assemblies, by cutting 13 nt before the 5' hit and 13 nt after the 3' hit, since this region (called the pri-extension region of the hairpin) was recently shown to have this average length in plants [35]. In order to predict the secondary structure of the precursors, the software mfold 3.2, free available at http://mfold.bioinfo.rpi.edu/cgi-bin/rna-form1.cgi, was used [36]. The minimal folding free energy index (MFEI) and the GC content were calculated for each sequence.

All the sequences with a MFEI greater than 0.85 were considered potential miRNA precursors [37]; besides, only 4 mismatches were allowed between the mature sequence and the passenger sequence, and only few and small asymmetric bulges were accepted [38].

### Identification of SNPs/indels at miRNA target sites

Polymorphisms in target genes have been searched through a comparison of the ESTs belonging to the same Unigene cluster. Each cluster has been assembled by Sequencer Software (Gene Codes) and polymorphisms have been searched on miRNA complementary sequence sites.

AutoSNP database http://autosnpdb.qfab.org.au was also screened using target gene annotations as contig-searching keywords.

## Authors' contributions

MC identified barley microRNAs and targets and highlighted the putative novel ones. He was also involved in polymorphism searching. PF conceived, designed and coordinated the work and wrote the manuscript. AS gave a contribution for polymorphisms searching and alignment of barley mature microRNA sequences. PB and AL prepared the additional material and gave a contribution to bioinformatics analysis. LC contributed in the design and discussion of the work and assisted in drafting the manuscript. All authors read and approved the final manuscript.

## Supplementary Material

Additional file 1 **BLAST results**. Alignments identified between plant miRNAs and barley ESTs. Click here for file

Additional file 2 **Barley mature miRNA sequences**. Aligned barley mature miRNA sequences grouped on the basis of the miRNA family. Families for which only one barley EST has been found to match are not reported in this file. Click here for file

Additional file 3 **microRNA matching ESTs**. List of ESTs matching the mature miRNA sequences. ESTs that present more than 1 target site for the same miRNA family or for different miRNA families have been highlighted. Click here for file

Additional file 4 **Secondary structures of the putative miRNA precursors**. Predicted structures of the identified putative miRNA precursors. The prediction was performed with mfold. Click here for file
