# Supplementary material for: A computational-based update on microRNAs and their targets in barley (Hordeum vulgare L.)
Source: BMC Genomics. 2010 Oct 22;11:595. doi: 10.1186/1471-2164-11-595 (PMC3091740; doi:10.1186/1471-2164-11-595)
Supplement: Additional file 2 — Barley mature miRNA sequences. Aligned barley mature miRNA sequences grouped on the basis of the miRNA family. Families for which only one barley EST has been found to match are not reported in this file. [file 1471-2164-11-595-S2.DOC]

- ***CLUSTAL 2.0.12 multiple sequence alignment miR156***

gb|EX597080.1| CTGACAGAAGAGAGAGAGCAC- 21

gb|BE060620.2| CTGACAGAAGAGAGAGAGCAC- 21

gb|EX597081.1 CTGACAGAAGAGAGAGAGCAC- 21

gb|FD527301.1| CTGACAGAAGAGAGAGAGCAC- 21

gb|FD528446.1| CTGACAGAAGAGAGAGAGCAC- 21

gb|GH218557.1| CTGACAGAAGAGAGAGAGCAC- 21

dbj|AV909109.1| -TGACAGAAGAGAGAGAGCA-- 19

dbj|AV910992.1| -TGACAGAAGAGAGAGAGCA-- 19

dbj|BJ485673.1| -TGACAGAAGAGAGAGAGCA-- 19

gb|BQ665502.1| -TGACAGAAGAGAGAGAGCA-- 19

gb|FD526024.1| -TGACAGAAGAGAGAGAGCACA 21

gb|EX598089.1| -TGACAGAAGAGAGAGAGCACA 21

gb|CA032492.1| -TGACAGAAGAGAGAGAGCACA 21

gb|CA020690.1| -TGACAGAAGAGAGAGAGCACA 21

dbj|BJ478354.1| -TGACAGAAGAGAGAGAGCACA 21

gb|GH228608.1| -TGACAGAAGAGAGAGAGCA-- 19

gb|BF258419.2| TTGACAGAAGAGAGAGAGCAC- 21

gb|EX576980.1| -TGACAGAAGAGAGGGAGCA-- 19

gb|BF630636.2| -TGACAGAAGAGAGGGAGC--- 18

gb|EX593949.1| -TGACAGAAGAGAGGGAGCA-- 19

gb|CB858958.1| -TGACAGAAGAGAGGGAGCA-- 19

gb|BG300360.1| -TGACAGAAGAGAGTGAGCAC- 20

- ***CLUSTAL 2.0.12 multiple sequence alignment miR157***

gb|CA032492.1| TTGACAGAAGAGAGAGAGCAC 21

gb|BF258419.2| TTGACAGAAGAGAGAGAGCAC 21

- ***CLUSTAL 2.0.12 multiple sequence alignment miR159***

emb|AJ480356.1| -TTGGAGTGAAGGGAGCTCC- 19

emb|AJ480355.1| -TTGGAGTGAAGGGAGCTCC- 19

gb|BQ466150.1| -TTGGAGTGAAGGGAGCTCC- 19

gb|CA006699.1| -TTGGAGTGAAGGGAGCTCC- 19

dbj|BJ448559.1| -TTGGATTGAAGGGAGCTC-- 18

dbj|BJ456281.1| -TTGGATTGAAGGGAGCTC-- 18

emb|AJ475696.1| -TTGGATTGAAGGGAGCTC-- 18

gb|AW983226.2| TTTGGATTGAGAGGAGCTCTT 21

- ***CLUSTAL 2.0.12 multiple sequence alignment miR160***

gb|CA002787.1 TGCCTGGCTCCCTGTATGCCA 21

gb|GH209508.1| TGCCTGGCTCCCTGTATGCC- 20

gb|BU986263.1| TGCCTGGCTCCCTGTATGCC- 20

dbj|BJ472971.1| TGCCTGGCTCCCTGTATGCC- 20

dbj|BJ471957.1 TGCCTGGCTCCCTGTATGCC- 20

dbj|BJ471544.1| TGCCTGGCTCCCTGTATGCC- 20

dbj|BJ469735.1 TGCCTGGCTCCCTGTATGCC- 20

dbj|BJ469445.1| TGCCTGGCTCCCTGTATGCC- 20

dbj|AV934494.1| TGCCTGGCTCCCTGTATGCC- 20

dbj|AV934488.1| TGCCTGGCTCCCTGTATGCC- 20

dbj|AV933114.1| TGCCTGGCTCCCTGTATGCC- 20

dbj|AV932125.1| TGCCTGGCTCCCTGTATGCC- 20

gb|BF622307.1 TGCCTGGCTCCCTGTATGC-- 19

gb|BF622299.2| TGCCTGGCTCCCTGTATGCC- 20

- ***CLUSTAL 2.0.12 multiple sequence alignment miR164***

gb|CB864307.1| TGGAGAAGCAGGGCACTTGCT 21

gb|BU991009.1| TGGAGAAGCAGGGCACTTGCT 21

gb|CK565851.1| TGGAGAAGCAGGGCACTTGCT 21

gb|CK566882.1| TGGAGAAGCAGGGCACTTGCT 21

gb|DN180933.1| TGGAGAAGCAGGGCACTTGCT 21

gb|DN187448.1| TGGAGAAGCAGGGCACTTGCT 21

gb|EX573211.1| TGGAGAAGCAGGGCACTTGCT 21

gb|EX573215.1| TGGAGAAGCAGGGCACTTGCT 21

gb|FD526177.1| TGGAGAAGCAGGGCACTTGCT 21

dbj|AV836807.1| TGGAGAAGCAGGTCACGTGCG 21

gb|BF256424.2| TGGAGAAGCAGGTCACGTGCG 21

emb|AL503516.1| TGGAGAAGCAGGTCACGTGCG 21

- ***CLUSTAL 2.0.12 multiple sequence alignment miR165***

dbj|BY841849.1| TCGGACCAGGCTTCATTCCCC 21

gb|BQ760548.1| TCGGACCAGGCTTCATTCCCC 21

- ***CLUSTAL 2.0.12 multiple sequence alignment miR166***

dbj|BY841849.1| CGGACCAGGCTTCATTCCC 19

gb|BQ760548.1| CGGACCAGGCTTCATTCCC 19

- ***CLUSTAL 2.0.12 multiple sequence alignment miR168***

gb|CA030148.1| GGCTTGGTGCAGCTCGGGAA 20

emb|AL508237.1| -GCTTGGTGCAGCTCGGGAA 19

gb|CA029324.1| GGCTTGGTGCAGCTCGGGAA 20

- ***CLUSTAL 2.0.12 multiple sequence alignment miR169***

gb|GH209055.1| AAGCCAAGGATGAGTTGCCTG 21

gb|EX600178.1| AAGCCAAGGATGAGTTGCCTG 21

gb|EX589523.1| AAGCCAAGGATGAGTTGCCTG 21

gb|DN188642.1| AAGCCAAGGATGAGTTGCCTG 21

gb|DN160484.1| AAGCCAAGGATGAGTTGCCTG 21

dbj|BJ474459.1| AAGCCAAGGATGAGTTGCCTG 21

dbj|BJ469257.1| AAGCCAAGGATGAGTTGCCTG 21

dbj|BJ482409.1| AAGCCAAGGATGAATTGCC-- 19

gb|GH222471.1| -AGCCAAGAATGATTTGCC-- 18

gb|GH222470.1| -AGCCAAGAATGATTTGCC-- 18

dbj|AV832521.1| -AGCCAAGAATGATTTGCC-- 18

- ***CLUSTAL 2.0.12 multiple sequence alignment miR171***

gb|CA009309.1| ---TTGAGCCGTGCCAATATC 18

gb|BQ461013.1| TGATTGAGCCGCGCCAATATC 21

- ***CLUSTAL 2.0.12 multiple sequence alignment miR172***

gb|GH213313.1| AGAATCCTGATGATGCTGCAG 21

gb|DN188672.1| AGAATCCTGATGATGCTGCAG 21

gb|DN182702.1| AGAATCCTGATGATGCTGCAG 21

gb|CD662228.1| AGAATCCTGATGATGCTGCAG 21

gb|CB878060.1| AGAATCCTGATGATGCTGCAG 21

gb|CB872382.1| AGAATCCTGATGATGCTGCAG 21

gb|BQ464715.1| AGAATCCTGATGATGCTGCAG 21

dbj|BJ462299.1| AGAATCCTGATGATGCTGCAG 21

dbj|BJ457290.1| AGAATCCTGATGATGCTGCAG 21

dbj|BJ454494.1| AGAATCCTGATGATGCTGCAG 21

dbj|BJ451082.1| AGAATCCTGATGATGCTGCAG 21

dbj|BJ449870.1| AGAATCCTGATGATGCTGCAG 21

gb|BM816977.1| AGAATCCTGATGATGCTGCAG 21

gb|BI954904.1| AGAATCCTGATGATGCTGCAG 21

dbj|AV912081.1| AGAATCCTGATGATGCTGCAG 21

- ***CLUSTAL 2.0.12 multiple sequence alignment miR393***

gb|GH212603.1| TCCAAAGGGATCGCATTG 18

gb|CV055207.1| TCCAAAGGGATCGCATTG 18

gb|CB860441.1| TCCAAAGGGATCGCATTG 18

gb|CA004635.1| TCCAAAGGGATCGCATTG 18

gb|BU973213.1| TCCAAAGGGATCGCATTG 18

dbj|BJ461212.1| TCCAAAGGGATCGCATTG 18

dbj|BJ453671.1| TCCAAAGGGATCGCATTG 18

gb|BE603282.2| TCCAAAGGGATCGCATTG 18

emb|AJ432418.1| TCCAAAGGGATCGCATTG 18

- ***CLUSTAL 2.0.12 multiple sequence alignment miR394***

gb|EX600346.1| TTGGCATTCTGTCCACCTCC 20

gb|EX595259.1| TTGGCATTCTGTCCACCTCC 20

gb|CK566049.1| TTGGCATTCTGTCCACCTCC 20

gb|CB882077.1| TTGGCATTCTGTCCACCTCC 20

dbj|BJ460700.1| TTGGCATTCTGTCCACCTCC 20

dbj|AV927829.1| TTGGCATTCTGTCCACCTCC 20

gb|AW982846.2| TTGGCATTCTGTCCACCTCC 20

dbj|AV923121.1| TTGGCATTCTGTCCACCTCC 20

- ***CLUSTAL 2.0.12 multiple sequence alignment miR395***

gb|CB882813.1| ATGAAGTGCTTGGAGGAACTC 21

gb|BI957514.1| ATGAAGTGCTTGGAGGAACTC 21

gb|CA011141.1| ATGAAGTGCTTGGAGGAACTC 21

- ***CLUSTAL 2.0.12 multiple sequence alignment miR396***

dbj|AV925436.1| TCCACAGGCTTTCTTGAACTG 21

gb|EX583134.1| TCCACAGGCTTTCTTGAAC-- 19

gb|CB861140.1| TCCACAGGCTTTCTTGAAC-- 19

gb|CB860915.1| TCCACAGGCTTTCTTGAAC-- 19

gb|CA031383.1| TCCACAGGCTTTCTTGAAC-- 19

gb|CA029723.1| TCCACAGGCTTTCTTGAAC-- 19

gb|CA026441.1| TCCACAGGCTTTCTTGAAC-- 19

gb|BQ665746.1| TCCACAGGCTTTCTTGAAC-- 19

gb|BQ660731.1| TCCACAGGCTTTCTTGAAC-- 19

gb|BQ470551.1| TCCACAGGCTTTCTTGAAC-- 19

gb|BI958861.1| TCCACAGGCTTTCTTGAAC—- 19

gb|BE455467.3| TCCACAGGCTTTCTTGAAC-- 19

- ***CLUSTAL 2.0.12 multiple sequence alignment miR397***

gb|FD518508.1| TTGAGTGCAGCGTTGATG 18

gb|CA014459.1| TTGAGTGCAGCGTTGATG 18

gb|CA007960.1| TTGAGTGCAGCGTTGATG 18

gb|CA005607.1| TTGAGTGCAGCGTTGATG 18

gb|BU966899.1| TTGAGTGCAGCGTTGATG 18

gb|BU966762.1| TTGAGTGCAGCGTTGATG 18

gb|BQ665274.1| TTGAGTGCAGCGTTGATG 18

gb|BQ461815.1| TTGAGTGCAGCGTTGATG 18

gb|BG415888.2| TTGAGTGCAGCGTTGATG 18

gb|BM097863.1| TTGAGTGCAGCGTTGATG 18

- ***CLUSTAL 2.0.12 multiple sequence alignment miR408***

gb|GH218076.1 -TGCACTGCCTCTTCCCTG-- 18

gb|CB881899.1 -TGCACTGCCTCTTCCCTG-- 18

gb|BU995745.1| -TGCACTGCCTCTTCCCTG-- 18

gb|BU995231.1| -TGCACTGCCTCTTCCCTG-- 18

gb|BU976391.1| -TGCACTGCCTCTTCCCTG-- 18

gb|BQ768355.1| -TGCACTGCCTCTTCCCTG-- 18

gb|BM372993.2| -TGCACTGCCTCTTCCCTG-- 18

gb|BQ468842.1| -TGCACTGCCTCTTCCCTG-- 18

gb|BM817241.1| -TGCACTGCCTCTTCCCTG-- 18

gb|BG344510.1| -TGCACTGCCTCTTCCCTG-- 18

gb|GH227347.1| CTGCACTGCCTCTGCCCTGGC 21

dbj|BY860269.1| CTGCACTGCCTCTGCCCTGGC 21

gb|DN161029.1| CTGCACTGCCTCTGCCCTGGC 21

gb|DN159885.1| CTGCACTGCCTCTGCCCTGGC 21

gb|DN159365.1| CTGCACTGCCTCTGCCCTGGC 21

gb|CX627754.1| CTGCACTGCCTCTGCCCTGGC 21

gb|BU995995.1| CTGCACTGCCTCTGCCCTGGC 21

gb|BI956607.1| CTGCACTGCCTCTGCCCTGGC 21

emb|AL503077.1| CTGCACTGCCTCTGCCCTGGC 21

- ***CLUSTAL 2.0.12 multiple sequence alignment miR414***

gb|GH225226.1| -CATCCTCATCATCCTCGTCC 20

gb|BI950673.1| -CATCCTCATCATCCTCGTCC 20

gb|EX586491.1| TCATCCTCATCATCCTCG--- 18

gb|BQ754240.1 TCATCTTCATCATCCTCGTCC 21

gb|BQ461123.1| TCATCCTCATCATCCTCCTCC 21

gb|GH215882.1 --ATCCTCATCATCATCGTC- 18

gb|BQ766907.1| --ATCCTCATCATCATCGTC- 18

gb|FD522676.1 -CATCCTCATCATCATCGTCC 20

dbj|BJ483887.1| -CATCCTCATCATCATCGTCC 20

emb|AJ463398.1| -CATCCTCATCATCATCGTCC 20

gb|FD519920.1| TCGTCCTCATCATCATCGTCC 21

gb|BU977216.1| TCGTCCTCATCATCATCGTCC 21

emb|AJ484531.1| TCGTCCTCATCATCATCGTCC 21

emb|AJ484529.1| TCGTCCTCATCATCATCGTCC 21

emb|AJ484528.1| TCGTCCTCATCATCATCGTCC 21

gb|CB869782.1| TCTTCCTCATCATCATCGTCC 21

gb|FD521791.1| TCATCCTCATCGTCATCGTCC 21

dbj|BY872409.1| TCATCCTCATCGTCATCGTCC 21

gb|CX629594.1| TCATCCTCATCGTCATCGTCC 21

gb|CB863626.1| TCATCCTCATCGTCATCGTCC 21

emb|AJ465879.1| TCATCCTCATCGTCATCGTCC 21

emb|AL508523.1| TCATCCTCATCATCATCATCC 21

gb|BF624285.1 TCATCCTCATCATCATCATCC 21

emb|AL508687.1| TCATCCTCATCATCATCATCC 21

emb|AL508968.1| TCATCCTCATCATCATCATCC 21

gb|BF621312.2| TCATCCTCATCATCATCATCC 21

gb|BF621429.2| TCATCCTCATCATCATCATCC 21

gb|BF258740.2| TCATCCTCATCATCATCATCC 21

gb|BF259945.2| TCATCCTCATCATCATCATCC 21

gb|BF260907.2| TCATCCTCATCATCATCATCC 21

gb|BG343488.1| TCATCCTCATCATCATCATCC 21

gb|BG367250.1| TCATCCTCATCATCATCATCC 21

gb|BF065291.2| TCATCCTCATCATCATCATCC 21

gb|BG414387.1| TCATCCTCATCATCATCATCC 21

gb|BI947590.1| TCATCCTCATCATCATCATCC 21

gb|BI955239.1| TCATCCTCATCATCATCATCC 21

gb|AW982339.3| TCATCCTCATCATCATCATCC 21

gb|BG343970.2| TCATCCTCATCATCATCATCC 21

gb|BG344143.2| TCATCCTCATCATCATCATCC 21

gb|BE601725.3| TCATCCTCATCATCATCATCC 21

gb|BG414930.2| TCATCCTCATCATCATCATCC 21

dbj|AV912777.1| TCATCCTCATCATCATCATCC 21

dbj|AV913800.1| TCATCCTCATCATCATCATCC 21

dbj|AV913905.1| TCATCCTCATCATCATCATCC 21

dbj|AV915428.1| TCATCCTCATCATCATCATCC 21

dbj|AV921124.1| TCATCCTCATCATCATCATCC 21

dbj|AV921412.1| TCATCCTCATCATCATCATCC 21

dbj|AV921518.1| TCATCCTCATCATCATCATCC 21

dbj|AV937410.1| TCATCCTCATCATCATCATCC 21

dbj|AV937472.1| TCATCCTCATCATCATCATCC 21

dbj|AV937556.1| TCATCCTCATCATCATCATCC 21

dbj|AV941158.1 TCATCCTCATCATCATCATCC 21

dbj|AV942356.1| TCATCCTCATCATCATCATCC 21

emb|AJ432426.1| TCATCCTCATCATCATCATCC 21

emb|AJ464292.1| TCATCCTCATCATCATCATCC 21

emb|AJ480350.1| TCATCCTCATCATCATCATCC 21

gb|BQ458535.1| TCATCCTCATCATCATCATCC 21

gb|BQ458595.1| TCATCCTCATCATCATCATCC 21

gb|BQ459299.1| TCATCCTCATCATCATCATCC 21

gb|BQ459712.1| TCATCCTCATCATCATCATCC 21

gb|BQ460277.1| TCATCCTCATCATCATCATCC 21

gb|BQ462213.1| TCATCCTCATCATCATCATCC 21

gb|BQ464684.1| TCATCCTCATCATCATCATCC 21

gb|BQ466775.1| TCATCCTCATCATCATCATCC 21

gb|BQ469640.1| TCATCCTCATCATCATCATCC 21

gb|BM097682.2| TCATCCTCATCATCATCATCC 21

gb|BM377485.2| TCATCCTCATCATCATCATCC 21

gb|BI780402.2| TCATCCTCATCATCATCATCC 21

gb|BM375756.2| TCATCCTCATCATCATCATCC 21

gb|BM375782.2| TCATCCTCATCATCATCATCC 21

gb|BI779941.2| TCATCCTCATCATCATCATCC 21

gb|BM376248.2| TCATCCTCATCATCATCATCC 21

gb|BM098136.2 TCATCCTCATCATCATCATCC 21

gb|BM372712.2 TCATCCTCATCATCATCATCC 21

gb|BI779417.2| TCATCCTCATCATCATCATCC 21

gb|BQ759670.1| TCATCCTCATCATCATCATCC 21

gb|BQ759811.1| TCATCCTCATCATCATCATCC 21

gb|BQ766211.1| TCATCCTCATCATCATCATCC 21

gb|BU966857.1| TCATCCTCATCATCATCATCC 21

gb|BU968828.1| TCATCCTCATCATCATCATCC 21

gb|BU970710.1| TCATCCTCATCATCATCATCC 21

gb|BU972641.1| TCATCCTCATCATCATCATCC 21

gb|BU975413.1| TCATCCTCATCATCATCATCC 21

gb|BU980322.1| TCATCCTCATCATCATCATCC 21

gb|BU981639.1| TCATCCTCATCATCATCATCC 21

gb|BU983266.1 TCATCCTCATCATCATCATCC 21

gb|BU983589.1| TCATCCTCATCATCATCATCC 21

gb|BU983969.1| TCATCCTCATCATCATCATCC 21

gb|BU987653.1| TCATCCTCATCATCATCATCC 21

gb|BU989445.1| TCATCCTCATCATCATCATCC 21

gb|BU993145.1| TCATCCTCATCATCATCATCC 21

gb|BU996890.1| TCATCCTCATCATCATCATCC 21

gb|CA002325.1| TCATCCTCATCATCATCATCC 21

gb|CA002862.1| TCATCCTCATCATCATCATCC 21

gb|CA003748.1| TCATCCTCATCATCATCATCC 21

gb|CA004561.1| TCATCCTCATCATCATCATCC 21

gb|CA013167.1| TCATCCTCATCATCATCATCC 21

gb|CA017576.1| TCATCCTCATCATCATCATCC 21

gb|CA019260.1| TCATCCTCATCATCATCATCC 21

gb|CA021043.1| TCATCCTCATCATCATCATCC 21

gb|CA023131.1| TCATCCTCATCATCATCATCC 21

gb|CA023204.1| TCATCCTCATCATCATCATCC 21

gb|CA029835.1| TCATCCTCATCATCATCATCC 21

gb|CA030825.1| TCATCCTCATCATCATCATCC 21

gb|CA031192.1| TCATCCTCATCATCATCATCC 21

dbj|BJ543899.1| TCATCCTCATCATCATCATCC 21

dbj|BJ543981.1| TCATCCTCATCATCATCATCC 21

dbj|BJ549281.1| TCATCCTCATCATCATCATCC 21

dbj|BJ549562.1| TCATCCTCATCATCATCATCC 21

dbj|BJ549768.1| TCATCCTCATCATCATCATCC 21

dbj|BJ549806.1| TCATCCTCATCATCATCATCC 21

gb|CB858222.1| TCATCCTCATCATCATCATCC 21

gb|CB874465.1| TCATCCTCATCATCATCATCC 21

gb|CB875758.1| TCATCCTCATCATCATCATCC 21

gb|CB881094.1| TCATCCTCATCATCATCATCC 21

gb|CK123482.1| TCATCCTCATCATCATCATCC 21

gb|CK123902.1| TCATCCTCATCATCATCATCC 21

gb|CV059257.1| TCATCCTCATCATCATCATCC 21

gb|CV062354.1| TCATCCTCATCATCATCATCC 21

gb|CV062462.1| TCATCCTCATCATCATCATCC 21

gb|DN180462.1| TCATCCTCATCATCATCATCC 21

gb|FD522901.1| TCATCCTCATCATCATCATCC 21

gb|EX590142.1| TCATCTTCATCATCATCGTC- 20

gb|BQ825890.1| TCATCTTCATCATCATCG--- 18

gb|BU994327.1| TCATCTTCATCATCATCGTC- 20

gb|EX591334.1| -CATCTTCATCATCATCGT-- 18

gb|FD522834.1| --ATCTTCATCATCATCGTCA 19

dbj|BJ468040.1| --ATCTTCATCATCATCGTCA 19

gb|BG368160.1| --ATCTTCATCATCATCGTCA 19

gb|FD518768.1| TCTTCTTCATCATCATCGTCA 21

gb|FD517906.1| TCTTCTTCATCATCATCGTCA 21

dbj|AV929203.1| TCTTCTTCATCATCATCGTCA 21

dbj|AV929202.1| TCTTCTTCATCATCATCGTCA 21

dbj|AV924108.1| TCTTCTTCATCATCATCGTCA 21

gb|EX573483.1| TCATCATCATCATCATCGTCA 21

gb|BQ755505.1| TCATCATCATCATCATCGTCA 21

gb|BI956855.1| TCATCATCATCATCATCGTCA 21

gb|GH226731.1| TCATCTTCATCATCATCATCA 21

gb|GH222395.1| TCATCTTCATCATCATCATCA 21

gb|FD520280.1 TCATCTTCATCATCATCATCA 21

dbj|BY842221.1| TCATCTTCATCATCATCATCA 21

dbj|BY840446.1| TCATCTTCATCATCATCATCA 21

dbj|BY845149.1| TCATCTTCATCATCATCATCA 21

gb|CX626649.1| TCATCTTCATCATCATCATCA 21

gb|CB881378.1| TCATCTTCATCATCATCATCA 21

gb|CB880263.1| TCATCTTCATCATCATCATCA 21

dbj|AV930239.1| TCATCTTCATCATCATCATCA 21

dbj|AV918210.1| TCATCTTCATCATCATCATCA 21

gb|BI954758.1| TCATCTTCATCATCATCATCA 21

- ***CLUSTAL 2.0.12 multiple sequence alignment miR444***

gb|GH228935.1| TGCAGTTGCTGCCTCAAGCTT 21

gb|CX630154.1| -GCAGTTGCTGCCTCAAGCTT 20

gb|CB882722.1| TGCAGTTGCTGCCTCAAGCTT 21

gb|CA019616.1| TGCAGTTGCTGCCTCAAGCTT 21

gb|CA008118.1| TGCAGTTGCTGCCTCAAGCTT 21

gb|CA006693.1| TGCAGTTGCTGCCTCAAGCTT 21

gb|BQ765702.1| TGCAGTTGCTGCCTCAAGCTT 21

gb|BQ765438.1| TGCAGTTGCTGCCTCAAGCTT 21

gb|BQ754066.1| TGCAGTTGCTGCCTCAAGCTT 21

emb|AL504006.1| TGCAGTTGCTGCCTCAAGCTT 21

gb|DN188095.1| TGCAGTTGCTGTCTCAAGCTT 21

gb|ca007403| TGCAGTTGTTGCCTCATGCTT 21

- ***CLUSTAL 2.0.12 multiple sequence alignment miR529***

gb|FD527301.1| AGAAGAGAGAGAGCACAGTCC 21

gb|BE060620.2| AGAAGAGAGAGAGCACAGTCC 21

gb|FD528446.1| AGAAGAGAGAGAGCACAGTCC 21

gb|GH218557.1| AGAAGAGAGAGAGCACAGTCC 21

dbj|BJ478354.1| AGAAGAGAGAGAGCACAG--- 18

gb|CA020690.1 AGAAGAGAGAGAGCACAG--- 18

gb|EX598089.1 AGAAGAGAGAGAGCACAG--- 18

gb|FD526024.1| AGAAGAGAGAGAGCACAG--- 18

gb|CA032492.1| AGAAGAGAGAGAGCACAGC-- 19

gb|BF258419.2| AGAAGAGAGAGAGCACAGC-- 19

- ***CLUSTAL 2.0.12 multiple sequence alignment miR818***

gb|EX600769.1| CCCTTATATTATGGGACGG 19

gb|CB863041.1| CCCTTATATTATGGGACGG 19

gb|CB862673.1| CCCTTATATTATGGGACGG 19

dbj|AV834592.1| CCCTTATATTATGGGACGG 19

dbj|AV832941.1| -CCTTATATTATGGGACGG 18

dbj|BJ482549.1| -CCTTATATTATGGGACGG 18

- ***CLUSTAL 2.0.12 multiple sequence alignment miR821***

gb|FD527963.1| TCAACAAAAAAGTTGAAT 18

dbj|BY868870.1| TCAACAAAAAAGTTGAAT 18

dbj|BY868178.1| TCAACAAAAAAGTTGAAT 18

dbj|BY867111.1| TCAACAAAAAAGTTGAAT 18

gb|CX627884.1| TCAACAAAAAAGTTGAAT 18

gb|CK565751.1| TCAACAAAAAAGTTGAAT 18

gb|CB874555.1| TCAACAAAAAAGTTGAAT 18

gb|CB858633.1| TCAACAAAAAAGTTGAAT 18

gb|CA015859.1| TCAACAAAAAAGTTGAAT 18

gb|BI777511.2| TCAACAAAAAAGTTGAAT 18

emb|AJ435431.1| TCAACAAAAAAGTTGAAT 18

dbj|AV835884.1| TCAACAAAAAAGTTGAAT 18

emb|AL503071.1| TCAACAAAAAAGTTGAAT 18

gb|BE422217.1| TCAACAAAAAAGTTGAAT 18

emb|AL502447.1| TCAACAAAAAAGTTGAAT 18

- ***CLUSTAL 2.0.12 multiple sequence alignment miR827***

gb|GH217971.1| TTAGATGACCATCAACGAA 19

gb|AW983262.2| TTAGATGACCATCAACGAA 19

dbj|BJ470052.1| TTAGATGACCATCAACGAA 19

- ***CLUSTAL 2.0.12 multiple sequence alignment miR1030***

dbj|BY853509.1| TCTCCATCTGCACCTGCACCA 21

dbj|BY852800.1| TCTCCATCTGCACCTGCACCA 21

dbj|BY852282.1| TCTCCATCTGCACCTGCACCA 21

dbj|BY851294.1| TCTCCATCTGCACCTGCACCA 21

dbj|BY849751.1| TCTCCATCTGCACCTGCACCA 21

dbj|BY849424.1| TCTCCATCTGCACCTGCACCA 21

gb|BQ470201.1| TCTCCATCTGCACCTGCACCA 21

gb|BF617675.2| TCTCCATCTGCACCTGCACCA 21

gb|BU997080.1| TCTCCATCTGCACCTGCACCA 21

emb|AJ462964.1 -CTGCATCTGCACCTGCAC-- 18

gb|BQ758674.1| -CTGCATCTGCACCTGCAC-- 18

gb|CX632992.1| -CTGCATCTGCACCTGCAC-- 18

gb|FD525982.1| CCTGCACCTGCACCTGCACCA 21

gb|CX627717.1| CCTGCACCTGCACCTGCACCA 21

gb|CB868672.1| CCTGCACCTGCACCTGCACCA 21

emb|AJ475105.1| CCTGCACCTGCACCTGCACCA 21

emb|AJ475104.1| CCTGCACCTGCACCTGCACCA 21

emb|AJ475103.1| CCTGCACCTGCACCTGCACCA 21

emb|AJ474302.1| CCTGCACCTGCACCTGCACCA 21

gb|BE196524.1| CCTGCACCTGCACCTGCACCA 21

- ***CLUSTAL 2.0.12 multiple sequence alignment miR1099***

gb|GH220133.1| TAGCAATGGTGTTTTTGT 18

gb|BG366282.2| TAGCAATGGTGTTTTTGT 18

gb|BU982260.1| TAGCAATGGTGTTTTTGT 18

- ***CLUSTAL 2.0.12 multiple sequence alignment miR1118***

gb|DN159500.1| CACCACATTGTGGAATGGAGGGA 23

dbj|BJ470538.1| CACTACATTGTGGTATGGAGGGA 23

- ***CLUSTAL 2.0.12 multiple sequence alignment miR1119***

gb|BE413256.1| TGGCACGGCGCGATGCTCAGTCAG 24

gb|BU967368.1| TGGCACGGCGCGATGCTCAGTCA- 23

emb|AL501165.1| TGGCACGGCGCGATGCTCAGTCAG 24

emb|AL501654.1| TGGCACGGCGCGATGCTCAGTCAG 24

emb|AL502875.1| TGGCACGGCGCGATGCTCAGTCAG 24

emb|AL503002.1| TGGCACGGCGCGATGCTCAGTCAG 24

emb|AL504115.1| TGGCACGGCGCGATGCTCAGTCAG 24

emb|AL505469.1| TGGCACGGCGCGATGCTCAGTCAG 24

emb|AL508350.1| TGGCACGGCGCGATGCTCAGTCAG 24

gb|BE602255.2| TGGCACGGCGCGATGCTCAGTCAG 24

gb|BG417782.1| TGGCACGGCGCGATGCTCAGTCAG 24

gb|BI776735.1| TGGCACGGCGCGATGCTCAGTCAG 24

gb|BI946902.1| TGGCACGGCGCGATGCTCAGTCAG 24

gb|BI959039.1| TGGCACGGCGCGATGCTCAGTCAG 24

gb|BI959664.1| TGGCACGGCGCGATGCTCAGTCAG 24

gb|BI960270.1| TGGCACGGCGCGATGCTCAGTCAG 24

gb|BG342907.2| TGGCACGGCGCGATGCTCAGTCAG 24

gb|BG343309.2| TGGCACGGCGCGATGCTCAGTCAG 24

gb|BM443638.1| TGGCACGGCGCGATGCTCAGTCAG 24

gb|BM443806.1| TGGCACGGCGCGATGCTCAGTCAG 24

gb|BM444122.1| TGGCACGGCGCGATGCTCAGTCAG 24

gb|BM444358.1| TGGCACGGCGCGATGCTCAGTCAG 24

gb|BM445267.1| TGGCACGGCGCGATGCTCAGTCAG 24

emb|AJ486202.1| TGGCACGGCGCGATGCTCAGTCAG 24

emb|AJ486203.1| TGGCACGGCGCGATGCTCAGTCAG 24

gb|BQ462148.1| TGGCACGGCGCGATGCTCAGTCAG 24

gb|BQ467836.1| TGGCACGGCGCGATGCTCAGTCAG 24

gb|BQ134689.1| TGGCACGGCGCGATGCTCAGTCAG 24

gb|BQ660575.1| TGGCACGGCGCGATGCTCAGTCAG 24

gb|BQ660599.1| TGGCACGGCGCGATGCTCAGTCAG 24

gb|BQ661979.1| TGGCACGGCGCGATGCTCAGTCAG 24

gb|BQ665864.1| TGGCACGGCGCGATGCTCAGTCAG 24

gb|BM443814.2| TGGCACGGCGCGATGCTCAGTCAG 24

gb|BI781077.2| TGGCACGGCGCGATGCTCAGTCAG 24

gb|BM096491.2| TGGCACGGCGCGATGCTCAGTCAG 24

gb|BM096529.2| TGGCACGGCGCGATGCTCAGTCAG 24

gb|BM372821.2| TGGCACGGCGCGATGCTCAGTCAG 24

gb|BM372872.2| TGGCACGGCGCGATGCTCAGTCAG 24

gb|BM441507.2| TGGCACGGCGCGATGCTCAGTCAG 24

gb|BI776941.2| TGGCACGGCGCGATGCTCAGTCAG 24

gb|BI776712.2| TGGCACGGCGCGATGCTCAGTCAG 24

gb|BI776713.2| TGGCACGGCGCGATGCTCAGTCAG 24

gb|BQ756674.1| TGGCACGGCGCGATGCTCAGTCAG 24

gb|BQ756701.1| TGGCACGGCGCGATGCTCAGTCAG 24

gb|BQ760313.1| TGGCACGGCGCGATGCTCAGTCAG 24

gb|BQ761024.1| TGGCACGGCGCGATGCTCAGTCAG 24

gb|BQ761269.1| TGGCACGGCGCGATGCTCAGTCAG 24

gb|BU970318.1| TGGCACGGCGCGATGCTCAGTCAG 24

gb|BU971666.1| TGGCACGGCGCGATGCTCAGTCAG 24

gb|BU973607.1| TGGCACGGCGCGATGCTCAGTCAG 24

gb|BU973892.1| TGGCACGGCGCGATGCTCAGTCAG 24

gb|BU973972.1| TGGCACGGCGCGATGCTCAGTCAG 24

gb|BU975470.1| TGGCACGGCGCGATGCTCAGTCAG 24

gb|BU975947.1| TGGCACGGCGCGATGCTCAGTCAG 24

gb|BU986680.1| TGGCACGGCGCGATGCTCAGTCAG 24

gb|BU993977.1| TGGCACGGCGCGATGCTCAGTCAG 24

gb|BU994188.1| TGGCACGGCGCGATGCTCAGTCAG 24

gb|CA016083.1| TGGCACGGCGCGATGCTCAGTCAG 24

gb|CA017073.1| TGGCACGGCGCGATGCTCAGTCAG 24

gb|CA018263.1| TGGCACGGCGCGATGCTCAGTCAG 24

gb|CA018933.1| TGGCACGGCGCGATGCTCAGTCAG 24

gb|CA020594.1| TGGCACGGCGCGATGCTCAGTCAG 24

gb|CA025269.1| TGGCACGGCGCGATGCTCAGTCAG 24

gb|CA026736.1| TGGCACGGCGCGATGCTCAGTCAG 24

gb|CA027063.1| TGGCACGGCGCGATGCTCAGTCAG 24

gb|CA027359.1| TGGCACGGCGCGATGCTCAGTCAG 24

gb|CA028720.1| TGGCACGGCGCGATGCTCAGTCAG 24

gb|CA029265.1| TGGCACGGCGCGATGCTCAGTCAG 24

gb|CB858181.1| TGGCACGGCGCGATGCTCAGTCAG 24

gb|CB858345.1| TGGCACGGCGCGATGCTCAGTCAG 24

gb|CB858768.1| TGGCACGGCGCGATGCTCAGTCAG 24

gb|CB858914.1| TGGCACGGCGCGATGCTCAGTCAG 24

gb|CB859747.1| TGGCACGGCGCGATGCTCAGTCAG 24

gb|CB866608.1| TGGCACGGCGCGATGCTCAGTCAG 24

gb|CB867081.1| TGGCACGGCGCGATGCTCAGTCAG 24

gb|CB868369.1| TGGCACGGCGCGATGCTCAGTCAG 24

gb|CB868554.1| TGGCACGGCGCGATGCTCAGTCAG 24

gb|CB870604.1| TGGCACGGCGCGATGCTCAGTCAG 24

gb|CB871755.1| TGGCACGGCGCGATGCTCAGTCAG 24

gb|CB871762.1| TGGCACGGCGCGATGCTCAGTCAG 24

gb|CB872108.1| TGGCACGGCGCGATGCTCAGTCAG 24

gb|CB872255.1| TGGCACGGCGCGATGCTCAGTCAG 24

gb|CB872284.1| TGGCACGGCGCGATGCTCAGTCAG 24

gb|CB872453.1| TGGCACGGCGCGATGCTCAGTCAG 24

gb|CB872600.1| TGGCACGGCGCGATGCTCAGTCAG 24

gb|CB874075.1| TGGCACGGCGCGATGCTCAGTCAG 24

gb|CB874191.1| TGGCACGGCGCGATGCTCAGTCAG 24

gb|CB874459.1| TGGCACGGCGCGATGCTCAGTCAG 24

gb|CB874734.1| TGGCACGGCGCGATGCTCAGTCAG 24

gb|CB874935.1| TGGCACGGCGCGATGCTCAGTCAG 24

gb|CB875296.1| TGGCACGGCGCGATGCTCAGTCAG 24

gb|CB876132.1| TGGCACGGCGCGATGCTCAGTCAG 24

gb|CB880289.1| TGGCACGGCGCGATGCTCAGTCAG 24

gb|CB880638.1| TGGCACGGCGCGATGCTCAGTCAG 24

gb|CB881890.1| TGGCACGGCGCGATGCTCAGTCAG 24

dbj|BY860649.1| TGGCACGGCGCGATGCTCAGTCAG 24

dbj|BY870666.1| TGGCACGGCGCGATGCTCAGTCAG 24

dbj|BY861857.1| TGGCACGGCGCGATGCTCAGTCAG 24

dbj|BY872818.1| TGGCACGGCGCGATGCTCAGTCAG 24

dbj|BY873695.1| TGGCACGGCGCGATGCTCAGTCAG 24

dbj|BY873820.1| TGGCACGGCGCGATGCTCAGTCAG 24

dbj|BY864489.1| TGGCACGGCGCGATGCTCAGTCAG 24

dbj|BY874148.1| TGGCACGGCGCGATGCTCAGTCAG 24

gb|FD525157.1| TGGCACGGCGCGATGCTCAGTCAG 24

gb|FD525832.1| TGGCACGGCGCGATGCTCAGTCAG 24

gb|FD527818.1| TGGCACGGCGCGATGCTCAGTCAG 24

gb|GH223037.1| TGGCACGGCGCGATGCTCAGTCAG 24

gb|GH223038.1| TGGCACGGCGCGATGCTCAGTCAG 24

gb|GH228802.1| TGGCACGGCGCGATGCTCAGTCAG 24

gb|GH227471.1| TGGCACGGCGCGATGCTCAGTCAG 24

gb|CB858500.1| -GGCACGGCGCGATGCTCAGTCAG 23

- ***CLUSTAL 2.0.12 multiple sequence alignment miR1120***

gb|CD054149.1| --ATTCTTATATTATGAAACGGAG 22

gb|CK566710.1| ACACTCTTATATTATGAAACGGA- 23

dbj|BY877161.1| ------TTATATTATGAGACGGAG 18

gb|EX584378.1| ACATTCTTATATTATGGGACAGAG 24

emb|AJ461536.1| ACATTCTTATATTATGGGACAGAG 24

dbj|BJ486588.1| ACATTCTTATATTATGGGACGGAG 24

- ***CLUSTAL 2.0.12 multiple sequence alignment miR1121***

dbj|BJ462560.1| ----GTGATCTAAACGCTCTTA 18

dbj|AV934721.1| ----GTGATCTAAACGCTCTTA 18

gb|BM443417.2| ----GTGATCTAAACGCTCTTA 18

gb|CX628296.1| ----GTGATCTAAACGCTCTTA 18

gb|FD524111.1| ---AGTGATCTAAACGCTCTTA 19

gb|BE216561.2| --TAGTGATCTAAACGCTCTTA 20

dbj|AV836141.1| --TAGTGATCTAAACGCTCTTA 20

gb|BI956127.1| --TAGTGATCTAAACGCTCTTA 20

dbj|AV910077.1| --TAGTGATCTAAACGCTCTTA 20

dbj|AV912245.1| --TAGTGATCTAAACGCTCTTA 20

dbj|AV918531.1| --TAGTGATCTAAACGCTCTTA 20

dbj|AV927890.1| --TAGTGATCTAAACGCTCTTA 20

dbj|AV928265.1| --TAGTGATCTAAACGCTCTTA 20

dbj|BJ447578.1| --TAGTGATCTAAACGCTCTTA 20

dbj|BJ448740.1| --TAGTGATCTAAACGCTCTTA 20

dbj|BJ455329.1| --TAGTGATCTAAACGCTCTTA 20

dbj|BJ461877.1| --TAGTGATCTAAACGCTCTTA 20

emb|AJ484963.1| --TAGTGATCTAAACGCTCTTA 20

emb|AJ484964.1| --TAGTGATCTAAACGCTCTTA 20

gb|BQ458760.1| --TAGTGATCTAAACGCTCTTA 20

gb|BQ656484.1| --TAGTGATCTAAACGCTCTTA 20

gb|BQ761197.1| --TAGTGATCTAAACGCTCTTA 20

gb|CK565957.1| --TAGTGATCTAAACGCTCTTA 20

gb|CK568591.1| --TAGTGATCTAAACGCTCTTA 20

gb|CK569040.1| --TAGTGATCTAAACGCTCTTA 20

gb|DN186686.1| --TAGTGATCTAAACGCTCTTA 20

gb|DN187056.1| --TAGTGATCTAAACGCTCTTA 20

gb|DN188870.1| --TAGTGATCTAAACGCTCTTA 20

dbj|BY867242.1| --TAGTGATCTAAACGCTCTTA 20

gb|EX587623.1| --TAGTGATCTAAACGCTCTTA 20

gb|EX587628.1| --TAGTGATCTAAACGCTCTTA 20

gb|EX601112.1| --TAGTGATCTAAACGCTCTTA 20

gb|EX601113.1| --TAGTGATCTAAACGCTCTTA 20

gb|EX591437.1| --TAGTGATCTAAACGCTCTTA 20

gb|EX591441.1| --TAGTGATCTAAACGCTCTTA 20

gb|FC555790.1| --TAGTGATCTAAACGCTCTTA 20

gb|FD522234.1| --TAGTGATCTAAACGCTCTTA 20

gb|GH208317.1| --TAGTGATCTAAACGCTCTTA 20

dbj|BY845018.1| AGTAGTGATCTAAACGCTCTTA 22

gb|CX630268.1| -GTAGTGATCTAAACGCTTTTA 21

gb|FD523912.1| AGTAGTGAGCTAAACGCTCTTA 22

gb|CA006505.1| AGTAGTGAGCTAAACGCTCTTA 22

gb|BU987786.1| AGTAGTGAGCTAAACGCTCTTA 22

dbj|BJ482407.1| AGTAGTGAGCTAAACGCTCTTA 22

gb|BM100883.1| AGTAGTGAGCTAAACGCTCTTA 22

gb|GH222673.1| AGTAGTGATCTAAATGCTCTTA 22

gb|FD518089.1| AGTAGTGATCTAAACACTCTTA 22

dbj|BY876909.1| AGTAGTGATCTAAACACTCTTA 22

dbj|BY875474.1| AGTAGTGATCTAAACACTCTTA 22

gb|BU974982.1| AGTAGTGATCTAAACACTCTTA 22

gb|EX599546.1| AGTAATGATCTAAACGCTCTTA 22

gb|EX599545.1| AGTAATGATCTAAACGCTCTTA 22

gb|CB866792.1| AGTAATGATCTAAACGCTCTTA 22

gb|EX586433.1| AGTAGTGATCTAAACGTTCTTA 22

gb|EX592001.1| AGTAGTGATCTAAACGTTCTTA 22

emb|AJ475374.1| AGTAGTGATCTAAACGATCTTA 22

dbj|BJ482541.1| AGTAGTGATTTAAACGCTCTTA 22

dbj|BY848300.1| AGTAGTGATCTAAACGCTCTTA 22

dbj|AV944995.1| --TAGTGATCTAAACGCTCTT- 19

gb|BM816024.1| --TAGTGATCTAAACGCTCTT- 19

gb|BM816030.1| --TAGTGATCTAAACGCTCTT- 19

dbj|BY840212.1| AGTAGTGATCTAAACGCTCTT- 21

dbj|BY847400.1| AGTAGTGATCTAAACGCTC--- 19

dbj|BY838250.1| AGTAGTGATCTAAACGCTCTT- 21

dbj|BY836959.1| AGTAGTGATCTAAACGCTCTT- 21

dbj|BY846289.1| AGTAGTGATCTAAACGCTCTT- 21

dbj|BY844680.1| AGTAGTGATCTAAACGCTCTT- 21

gb|BU970654.1| --TAGTGATCTAAACGCTCTT- 19

- ***CLUSTAL 2.0.12 multiple sequence alignment miR1122***

gb|FD518678.1| TAGATACATCCGTATCTAGA 20

gb|DN157625.1| TAGATACATCCGTATCTAGA 20

dbj|BY876819.1| TAGATACATCCGTATCTA-- 18

gb|EX593109.1| TAGATACATCCGTATCTA-- 18

- ***CLUSTAL 2.0.12 multiple sequence alignment miR1126***

gb|CA013466.1| TCCACTACGGACTACATACAGAG 23

gb|BQ740191.1| TCCACTACGTACTACATACGGAG 23

gb|EX581233.1| --CACTATGGACTACATACG--- 18

gb|EX581232.1| --CACTATGGACTACATACG--- 18

gb|EX594051.1| --CACTATGGACTACATACG--- 18

gb|CA012080.1| TCCACTATGGACTACATACGGA- 22

gb|BU974512.1| TCAACTATGGACTACATACGGA- 22

emb|AJ469590.1| --CACTATGGACTACATACGGA- 20

gb|CX629047.1| TCCACTATGGACTACATACGGAG 23

gb|BQ663275.1| --CATTATGGACTACATACGGAG 21

dbj|AV911540.1| --CACTATGGACTAAATACGGAG 21

dbj|BJ480160.1| TCCACTATGGACTACATACAGAG 23

gb|CA012079.1| TCCACTACAGACTACATACGGAG 23

gb|AF069328.1| TCCACTATAGACTAGATACGGAG 23

- ***CLUSTAL 2.0.12 multiple sequence alignment miR1128***

gb|GH225521.1| ---TACTCCCTCCGTCCGAAA 18

gb|GH224188.1| ---TACTCCCTCCGTCCGAAA 18

dbj|AV912559.1| ---TACTCCCTCCGTCCGAAA 18

gb|BE060539.3| ---TACTCCCTCCGTCCGAAA 18

dbj|BJ465876.1| ---TACTCCCTCCGTCCGAAA 18

gb|BQ465197.1| ---TACTCCCTCCGTCCGAAA 18

gb|BM368614.2| ---TACTCCCTCCGTCCGAAA 18

gb|BU978892.1| ---TACTCCCTCCGTCCGAAA 18

dbj|BJ546620.1| ---TACTCCCTCCGTCCGAAA 18

gb|CB865150.1| ---TACTCCCTCCGTCCGAAA 18

dbj|BY875862.1| ---TACTCCCTCCGTCCGAAA 18

gb|EX581997.1| ---TACTCCCTCCGTCCGAAA 18

gb|EX597992.1| ---TACTCCCTCCGTCCGAAA 18

gb|EX589384.1| ---TACTCCCTCCGTCCGAAA 18

gb|EX589385.1| ---TACTCCCTCCGTCCGAAA 18

gb|EX575082.1| ---TACTCCCTCCGTCCGAAA 18

gb|EX575083.1| ---TACTCCCTCCGTCCGAAA 18

gb|EX575273.1| ---TACTCCCTCCGTCCGAAA 18

gb|EX575274.1| ---TACTCCCTCCGTCCGAAA 18

gb|EX595629.1| ---TACTCCCTCCGTCCGAAA 18

gb|EX575438.1| TACTACTCCCTCCGTCCCAAA 21

gb|EX581151.1| TACTACTCCCTCCGTCCCAAA 21

gb|DN160816.1| TACTACTCCCTCCGTCCCAAA 21

dbj|BJ473839.1| TACTACTCCCTCCGTCCCAAA 21

emb|AJ436472.1| TACTACTCCCTCCGTCCCAAA 21

dbj|BY874640.1| TACTACTCCCTCCGTCCTAAA 21

gb|CB871794.1| TACTACTCCCTCCGTCCTAAA 21

gb|GH217363.1| TACTACTCCCTCCGTCCGGAA 21

gb|BI954805.1| TACTACTCCCTCCGTCCG--- 18

gb|FD523856.1| TACTACTCCCTCCGTCCGGAA 21

dbj|BY858979.1| TACTACTCCCTCCGTCCGGAA 21

gb|CX628102.1| TACTACTCCCTCCGTCCGGAA 21

gb|CX627027.1| TACTACTCCCTCCGTCCGGAA 21

gb|CV062707.1| TACTACTCCCTCCGTCCGGAA 21

- ***CLUSTAL 2.0.12 multiple sequence alignment miR1130***

gb|EX594898.1| CCTCTGTCTCATAATGTAAGACG 23

dbj|BJ474583.1| CCTCCGTTTCATAATGTAAGACG 23

gb|GH217963.1| CCTCCGTCGCATAATGTAAGACG 23

gb|BG344337.1| CCTCCGTCGCATAATGTAAGACG 23

gb|DN159362.1| CCTCCGTCCCATAATGTAAGACG 23

gb|BM100733.2| CCTCCGTCCCATAATGTAAGACG 23

gb|EX580538.1| CCTCCGTCTCATAATGTAAGACG 23

gb|EX580539.1| CCTCCGTCTCATAATGTAAGACG 23

gb|EX586705.1| CCTCCGTCTCATAATGTAAGACG 23

gb|CX626126.1| -CTCCGTCTCGCAATGTAAGACG 22

- ***CLUSTAL 2.0.12 multiple sequence alignment miR1133***

gb|FD527254.1| -ATATACTCCC-TCCGTCCCAAA 21

gb|EX598525.1| -ATATACTCCC-TCCGTCCCAAA 21

gb|EX598524.1| -ATATACTCCC-TCCGTCCCAAA 21

gb|EX584248.1| -ATATACTCCC-TCCGTCCCAAA 21

gb|EX584247.1| -ATATACTCCC-TCCGTCCCAAA 21

gb|EX583701.1| -ATATACTCCC-TCCGTCCCAAA 21

gb|EX583700.1| -ATATACTCCC-TCCGTCCCAAA 21

gb|EX587619.1| -ATATACTCCC-TCCGTCCCAAA 21

gb|EX587618.1| -ATATACTCCC-TCCGTCCCAAA 21

gb|EX590265.1| -ATATACTCCC-TCCGTCCCAAA 21

gb|EX590264.1| -ATATACTCCC-TCCGTCCCAAA 21

gb|CB861063.1| -ATATACTCCC-TCCGTCCCAAA 21

gb|GH218255.1| CATATACTCCC-TCCGTCCCAAA 22

gb|GH225521.1| ----TACTCCC-TCCGTCCGAAA 18

gb|BQ465197.1| -ATGTACTCCC-TCCGTCCGAAA 21

gb|GH224188.1| ----TACTCCC-TCCGTCCGAAA 18

gb|EX575083.1| ----TACTCCC-TCCGTCCGAAA 18

gb|EX575082.1| ----TACTCCC-TCCGTCCGAAA 18

gb|EX589385.1| ----TACTCCC-TCCGTCCGAAA 18

dbj|BJ465876.1| ----TACTCCC-TCCGTCCGAAA 18

gb|BM368614.2| ----TACTCCC-TCCGTCCGAAA 18

gb|CB865150.1| ----TACTCCC-TCCGTCCGAAA 18

gb|EX581997.1| ----TACTCCC-TCCGTCCGAAA 18

gb|EX597992.1| ----TACTCCC-TCCGTCCGAAA 18

gb|EX589384.1| ----TACTCCC-TCCGTCCGAAA 18

gb|BE060539.3| ---ATACTCCC-TCCGTCCGAAA 19

dbj|AV912559.1| -ATATACTCCC-TCCGTCCGAAA 21

gb|BU978892.1| -ATATACTCCC-TCCGTCCGAAA 21

dbj|BJ546620.1| -ATATACTCCC-TCCGTCCGAAA 21

dbj|BY875862.1 CATATACTCCC-TCCGTCCGAAA 22

gb|EX575273.1| CATATACTCCC-TCCGTCCGAAA 22

gb|EX575274.1| CATATACTCCC-TCCGTCCGAAA 22

gb|DN155230.1| -ATATACTCCT-TCCGTCCGAAA 21

gb|EX584438.1| -ATATACTCCC-TCTGTCCGAAA 21

gb|EX584437.1| -ATATACTCCC-TCTGTCCGAAA 21

gb|EX595629.1| CATATACTCCC-TCCGTCCGAAA 22

gb|DN159362.1| CATATACTCCC-TCCGTCC---- 18

gb|BU991944.1| CATATACTCCC-TCCGTCC---- 18

dbj|BJ545549.1| CATATACTNCCCTCCGTCCGAAA 23

gb|CK568133.1| CATATACTCCC-TCCGTCCGGAA 22

gb|EX582390.1| -ATATACTCCC-TCCGTCCGGAA 21

gb|EX582389.1| -ATATACTCCC-TCCGTCCGGAA 21

- ***CLUSTAL 2.0.12 multiple sequence alignment miR1134***

gb|BQ762321.1| ---CAACAACAAGAAGAAGAAGAT-- 21

gb|BM443632.2| ---CAACAACAAGAAGAGGAAGAT-- 21

gb|BF259853.3| ---CAACAACAAGAAGAAGAAGAT-- 21

gb|BF625278.1| ---CAACAACAAGAAGAAGAAGAT-- 21

gb|BF625738.2| ---CAACAACAAGAAGAAGAAGAT-- 21

gb|BF618754.2| ---CAACAACAAGAAGAAGAAGAT-- 21

gb|AW982841.2| ---CAACAACAAGAAGAAGAAGAT-- 21

gb|BI952369.1| ---CAACAACAAGAAGAAGAAGAT-- 21

gb|BI955167.1| ---CAACAACAAGAAGAAGAAGAT-- 21

gb|BG344997.2| ---CAACAACAAGAAGAAGAAGAT-- 21

gb|BQ762530.1| ---CAACAACAAGAAGAAGAAGAT-- 21

gb|BQ762580.1| ---CAACAACAAGAAGAAGAAGAT-- 21

gb|BU998858.1| ---CAACAACAAGAAGAAGAAGAT-- 21

gb|CA029064.1| ---CAACAACAAGAAGAAGAAGAT-- 21

gb|CB882389.1| ---CAACAACAAGAAGAAGAAGAT-- 21

gb|CK125154.1| ---CAACAACAAGAAGAAGAAGAT-- 21

dbj|BY844640.1| ---CAACAACAAGAAGAAGAAGAT-- 21

dbj|BY852895.1| ---CAACAACAAGAAGAAGAAGAT-- 21

dbj|BY841987.1| ---CAACAACAAGAAGAAGAAGAT-- 21

gb|GH217107.1| ---CAACAACAAGAAGAAGAAGAT-- 21

gb|GH226934.1| ---CAACAACAAGAAGAAGAAGAT-- 21

gb|BI777307.2| -AAGAACAACATGAAGAAGAAGAT-- 23

dbj|BY841026.1| CAACAACA-CAAGAAGAAGAAGA--- 22

dbj|BY846186.1| CAACAACA-CAAGAAGAAGAAGA--- 22

gb|CB859022.1| CACCAACACCAAGAAGAAGAAGA--- 23

gb|BQ762766.1| CACCAACACCAAGAAGAAGAAGA--- 23

gb|BQ761195.1| CACCAACACCAAGAAGAAGAAGA--- 23

gb|BQ761146.1| CACCAACACCAAGAAGAAGAAGA--- 23

emb|AL499742.1| CACCAACACCAAGAAGAAGAAGA--- 23

gb|CV063154.1| CAGCAACAACAGGAAGAAGAAGA--- 23

gb|CV062535.1| CAGCAACAACAGGAAGAAGAAGA--- 23

gb|CV060319.1| CAGCAACAACAGGAAGAAGAAGA--- 23

gb|BU976670.1| -AACAAGAACAAGAAGAAGAAGA--- 22

dbj|BJ454017.1| -AACAAGAACAAGAAGAAGAAG---- 21

gb|BQ459561.1 -AACAAGAACAAGAAGAAGAAGA--- 22

dbj|AV832512.1| -AACAAGAACAAGAAGAAGAAGA--- 22

gb|BI956461.1| ---CAAGAACAAGAAGAAGAAGAAGA 23

gb|BG300343.1| ---CAAGAACAAGAAGAAGAAGAAGA 23

gb|EX591891.1| CAAAAACAACAAGAAGAAGAA----- 21

gb|EX591890.1| CAAAAACAACAAGAAGAAGAA----- 21

gb|CK569529.1| CAAAAACAACAAGAAGAAGAA----- 21

gb|CK566900.1| CAAAAACAACAAGAAGAAGAA----- 21

gb|GH227222.1| -AACAACAACAAGAAGAAGAA----- 20

gb|CA020517.1| CAACAACAACAAGAGGAAGAAG---- 22

gb|BM100994.2| CAACAACAACAAGAGGAAGAAG---- 22

dbj|BY858111.1| CAACAACAACAACAAGAAGAAG---- 22

dbj|BY857525.1| CAACAACAACAACAAGAAGAAG---- 22

dbj|BY855942.1| CAACAACAACAACAAGAAGAAG---- 22

dbj|BY855822.1| CAACAACAACAACAAGAAGAAG---- 22

gb|BM371389.2| CAACAACAACAACAACAAGAAGA--- 23

gb|GH216071.1| CAACAACAACAA-AAGAAGAAGA--- 22

gb|CB876062.1| CAACAACAACAA-AAGAAGAAGA--- 22

gb|CB869817.1| CAACAACAACAA-AAGAAGAAGA--- 22

gb|BQ762093.1| CAACAACAACAA-AAGAAGAAGA--- 22

gb|BQ754930.1| CAACAACAACAA-AAGAAGAAGA--- 22

gb|BQ665064.1| CAACAACAACAA-AAGAAGAAGA--- 22

gb|BQ470166.1| CAACAACAACAA-AAGAAGAAGA--- 22

gb|GH220319.1| CAACAACGACAAGAAGAAGAAGA--- 23

gb|BQ739939.1| CAACAACGACNAGAAGAAGAAGA--- 23

dbj|BY840872.1| CAACAACGACAAGAAGAAGAAGA--- 23

gb|CA014084.1| CAACAACGACAAGAAGAAGAAGA--- 23

gb|BU987552.1| CAACAACGACAAGAAGAAGAAGA--- 23

gb|BM369234.2| CAACAACGACAAGAAGAAGAAGA--- 23

gb|BI779896.2| CAACAACGACAAGAAGAAGAAGA--- 23

gb|BG369020.1| CAACAACGACAAGAAGAAGAAGA--- 23

gb|BG366730.1| CAACAACGACAAGAAGAAGAAGA--- 23

gb|BU989003.1| CAACAACAACAAGAAGAAGAAGA--- 23

gb|GH207905.1| -AACAACAACAAGAAGAAG------- 18

gb|BG309315.2| -AACAACAACAAGAAGAAG------- 18

gb|BE215081.1| -AACAACAACAAGAAGAAG------- 18

dbj|BJ454171.1| -AACAACAACAAGAAGAAGA------ 19

- ***CLUSTAL 2.0.12 multiple sequence alignment miR1135***

dbj|AV921170.1| -TGCGATAAGTAATTCCGGACGGA 23

dbj|AV920708.1| -TGCGATAAGTAATTCCGGACGGA 23

dbj|BJ476131.1| -TGCGATAAGTAATTCCGGACGGA 23

gb|EX597422.1| -TGCGATAAGTAATTCCGGACGGA 23

gb|EX597427.1| -TGCGATAAGTAATTCCGGACGGA 23

gb|FD523444.1| -TGCGATAAGTAATTCCGGACGGA 23

gb|EX595506.1| -TGCGACAAGTAATTCCTGACGGA 23

gb|EX595505.1| -TGCGACAAGTAATTCCTGACGGA 23

gb|FD517994.1| -TGTGACAAGTAATTCCGGACGGA 23

gb|DN180267.1| -TGTGACAAGTAATTCCGGACGGA 23

gb|CA016582.1| -TGTGACAAGTAATTCCGGACGGA 23

gb|BU991683.1| -TGTGACAAGTAATTCCGGACGGA 23

gb|BU973936.1| -TGTGACAAGTAATTCCGGACGGA 23

dbj|BY858979.1| -TGGGACAAGTAATTCCGGACGGA 23

gb|CX628102.1| -TGGGACAAGTAATTCCGGACGGA 23

gb|CV062707.1| -TGGGACAAGTAATTCCGGACGGA 23

gb|CA010201.1| -TGCGACAAGTAATTCCGGACGGA 23

gb|BI954805.1| -TGCGACAAGTAATTCCGGATGGA 23

gb|FD524148.1| -TGCGACAAGTAATTCCGAA---- 19

gb|DN184813.1| -TGCGACAAGTAATTCCGAA---- 19

emb|AJ462658.1| -TGCGACAAGTAATTCCGAA---- 19

dbj|AV834372.1| -TGCGACAAGTAATTCCGAA---- 19

gb|EX598506.1| CTGCGACAAGTAATTCCGGACG-- 22

emb|AL450817.1| CTGCGACAAGTAATTCCGGACNGA 24

- ***CLUSTAL 2.0.12 multiple sequence alignment miR1136***

dbj|BJ546620.1| TTGTCGAAGGAATGGATGTATCTA 24

dbj|BJ545549.1| TTGTCGAAGGAATGGATGTATCTA 24

dbj|AV912559.1| TTGTCGAAGGAATGGATGTATCTA 24

gb|BU978892.1| TTGTCGGAGGAATGGATGTATCTA 24

- ***CLUSTAL 2.0.12 multiple sequence alignment miR1137***

gb|EX595424.1| TAGTACAAAGTTGAGTCA 18

gb|EX595419.1| TAGTACAAAGTTGAGTCA 18

dbj|BJ475341.1| TAGTACAAAGTTGAGTCA 18

dbj|BJ474657.1 TAGTACAAAGTTGAGTCA 18

dbj|BJ474050.1| TAGTACAAAGTTGAGTCA 18

dbj|AV936793.1| TAGTACAAAGTTGAGTCA 18

dbj|BJ473864.1| TAGTACAAAGTTGAGTCA 18

- ***CLUSTAL 2.0.12 multiple sequence alignment miR1171***

dbj|BY849810.1| TGGAGTGGAGTGGAGTGG----- 18

dbj|BY844443.1| TGGAGTGGAGTGGAGTGG----- 18

bm373074.2 TGGAGTGGAGTGGAGTGGA---- 19

gb|BQ468092.1 TGGAGTGGAGTGGAGTGGA---- 19

gb|BF268131.3| TGGAGTGGAGTGGAGTGGAGT-- 21

dbj|BY857362.1| TGGAGTGGAGTGGAGTGGAGTG- 22

gb TGGAGTGGAGTGGAGTGGAGTGG 23

bq468092.1 TGGAGTGGAGTGGAGTGGAGTGG 23

gb|BE060388.3| TGGAGTGGAGTGGAGTGGAGTGG 23

dbj|BY849616.1| -GGAGTGGAGTGGAGTGGAG--- 19

dbj|BY843802.1| -GGAGTGGAGTGGAGTGGAG--- 19

dbj|BY841512.1| -GGAGTGGAGTGGAGTGGAG--- 19

gb|BG343039.2| --GAGTGGAGTGGAGTGGAG--- 18

gb|BG343050.2| --GAGTGGAGTGGAGTGGAG--- 18

gb|BQ760964.1| --GAGTGGAGTGGAGTGGAG--- 18

dbj|BY849250.1| --GAGTGGAGTGGAGTGGAG--- 18

gb|GH216700.1| --GAGTGGAGTGGAGTGGAG--- 18

gb|AW983298.2| TGGAGTGGAGTGGAGTGGAGTGG 23

gb|BQ463190.1 TGGAGTGGAGTGGAGTGGAGTGG 23

gb|BF625321.2| -GGAGTGGAGTGGAGTGGAGTGG 22

gb|GH210205.1| -GGAGTGGAGTGGAGTGGAGTGG 22

gb|GH206880.1| -GGAGTGGAGTGGAGCGGAGTGG 22

gb|GH218429.1| --GAGTGGAGTGGAGTGGAGTGG 21

gb|CA025249.1 --GAGTGGAGGGGAGTGGAGTGG 21

gb|BU980904.1| --GAGTGGAGGGGAGTGGAGTGG 21

gb|BU967787.1| --GAGTGGAGGGGAGTGGAGTGG 21

gb|BU967712.1| --GAGTGGAGGGGAGTGGAGTGG 21

- ***CLUSTAL 2.0.12 multiple sequence alignment miR1436***

gb|GH219034.1| --ATTATGGGACGGAGGGAGT 19

gb|EX581160.1| --ATTATGGGACGGAGGGAG- 18

gb|FD519329.1| --ATTATGGGACGGAGGGAGT 19

emb|AL503060.1| ---TTATGGGACGGAGGGAGT 18

emb|AL500558.1| ---TTATGGGACGGAGGGAGT 18

emb|AL509815.1| ---TTATGGGACGGAGGGAGT 18

dbj|AV834234.1| ---TTATGGGACGGAGGGAGT 18

dbj|BJ486097.1| ---TTATGGGACGGAGGGAGT 18

gb|BQ467319.1| ---TTATGGGACGGAGGGAGT 18

gb|BQ468272.1| ---TTATGGGACGGAGGGAGT 18

gb|BQ657747.1| ---TTATGGGACGGAGGGAGT 18

gb|BQ661628.1| ---TTATGGGACGGAGGGAGT 18

gb|BQ661883.1| ---TTATGGGACGGAGGGAGT 18

gb|BQ662944.1| ---TTATGGGACGGAGGGAGT 18

gb|BU968160.1| ---TTATGGGACGGAGGGAGT 18

gb|BU977032.1| ---TTATGGGACGGAGGGAGT 18

gb|BU996201.1| ---TTATGGGACGGAGGGAGT 18

gb|CA010562.1| ---TTATGGGACGGAGGGAGT 18

gb|CB877969.1| ---TTATGGGACGGAGGGAGT 18

gb|CX629150.1| ---TTATGGGACGGAGGGAGT 18

gb|DN180754.1| ---TTATGGGACGGAGGGAGT 18

gb|DN184585.1| ---TTATGGGACGGAGGGAGT 18

gb|DN187130.1| ---TTATGGGACGGAGGGAGT 18

dbj|BY870096.1| ---TTATGGGACGGAGGGAGT 18

dbj|BY863869.1| ---TTATGGGACGGAGGGAGT 18

dbj|BY864150.1| ---TTATGGGACGGAGGGAGT 18

gb|FD521311.1| ---TTATGGGACGGAGGGAGT 18

gb|GH213790.1| ---TTATGGGACGGAGGGAGT 18

gb|AW983316.2| --ATTATGGGACGGAGGGAGT 19

dbj|AV834592.1| --ATTATGGGACGGAGGGAGT 19

dbj|BJ468182.1| --ATTATGGGACGGAGGGAGT 19

dbj|BJ482549.1| --ATTATGGGACGGAGGGAGT 19

gb|BM371881.2| --ATTATGGGACGGAGGGAGT 19

gb|CA010088.1| --ATTATGGGACGGAGGGAGT 19

gb|CB862673.1| --ATTATGGGACGGAGGGAGT 19

gb|CB863041.1| --ATTATGGGACGGAGGGAGT 19

gb|CD057247.1| --ATTATGGGACGGAGGGAGT 19

gb|CD663278.1| --ATTATGGGACGGAGGGAGT 19

gb|CK569835.1| --ATTATGGGACGGAGGGAGT 19

gb|CV064340.1| --ATTATGGGACGGAGGGAGT 19

dbj|BY865147.1| --ATTATGGGACGGAGGGAGT 19

dbj|BY874438.1| --ATTATGGGACGGAGGGAGT 19

dbj|BY877744.1| --ATTATGGGACGGAGGGAGT 19

gb|EX600769.1| --ATTATGGGACGGAGGGAGT 19

gb|EX595552.1| --ATTATGGGACGGAGGGAGT 19

gb|EX595547.1| --ATTATGGGACGGAGGGAGT 19

gb|EX573766.1| --ATTATGGGACGGAGGGAGT 19

dbj|BJ485913.1| ACATTATGGGACGGAGGGAGT 21

dbj|BJ485923.1| ACATTATGGGACGGAGGGAGT 21

gb|BM100733.2| ACATTATGGGACGGAGGGAGT 21

gb|DN159362.1| ACATTATGGGACGGAGGGAGT 21

gb|EX572464.1| ACATTATGGGACGGAGGGAGT 21

gb|EX591500.1| ACATTTTGGGACGGAGGGAGT 21

dbj|BY874640.1| ACATTTTGGGACGGAGGGAGT 21

gb|CB871794.1| ACATTTTGGGACGGAGGGAGT 21

gb|CB867028.1| ACATTTTGGGACGGAGGGAGT 21

gb|EX593248.1| ACATTATGGGACGTAGGGAGT 21

gb|GH212963.1| ACATTATGGGACAGAGGGAGT 21

gb|EX583817.1| ACATTATGGGACAGAGGGAGT 21

gb|EX592251.1| ACATTATGGGACAGAGGGAGT 21

gb|EX592250.1| ACATTATGGGACAGAGGGAGT 21

gb|EX591321.1| ACATTATGGGACAGAGGGAGT 21

gb|BF629274.2| ACATTATGGGACAGAGGGAGT 21

gb|GH217963.1| ACATTATGCGACGGAGGGAGT 21

gb|BG344337.1| ACATTATGCGACGGAGGGAGT 21

gb|EX575535.1| ACATTATGTGACGGAGGGAGT 21

gb|EX583713.1| ACATTATGTGACGGAGGGAGT 21

gb|EX583708.1| ACATTATGTGACGGAGGGAGT 21

gb|EX593880.1| ACATTATGTGACGGAGGGAGT 21

gb|BM443538.2| ACATTATGTGACGGAGGGAGT 21

gb|EX586705.1| ACATTATGAGACGGAGGGAGT 21

gb|EX580539.1 ACATTATGAGACGGAGGGAGT 21

gb|EX580538.1| ACATTATGAGACGGAGGGAGT 21

- ***CLUSTAL 2.0.12 multiple sequence alignment miR1439***

gb|GH215029.1| TTTTGGAACGGAGTGAGTA-- 19

gb|BU984969.1| TTTTGGAACGGAGTGAGTA-- 19

dbj|BJ473266.1| TTTTGGAACGGAGTGAGTA-- 19

dbj|BJ472225.1| TTTTGGAACGGAGTGAGTA-- 19

dbj|BJ471375.1| TTTTGGAACGGAGTGAGTA-- 19

dbj|BJ470543.1| TTTTGGAACGGAGTGAGTA-- 19

gb|BF256174.1| TTTTGGAACGGAGTGAGTA-- 19

dbj|BJ486131.1| TTTAGGAACGGAGTGAGTATT 21

dbj|AV836203.1| TTTAGGAACGGAGTGAGTATT 21

dbj|BY874243.1| TTTTGGGACGGAGTGAGTATT 21

gb|BQ658389.1| TTTTGGGACGGAGTGAGTATT 21

gb|BQ658360.1| TTTTGGGACGGAGTGAGTATT 21

dbj|BY863662.1| TTTTGGAACGGAGGGAGTATT 21

gb|CD664034.1| TTTTGGAACGGAGAGAGTATT 21

- ***CLUSTAL 2.0.12 multiple sequence alignment miR1533***

gb|EX591095.1| TAATAAAAATAATAATGA 18

gb|EX582095.1| TAATAAAAATAATAATGA 18

gb|EX582096.1| TAATAAAAATAATAATGA 18

- ***CLUSTAL 2.0.12 multiple sequence alignment miR1867***

gb|GH216548.1| -----TTTCTAGGACAG-AGGGAG- 18

gb|BF260484.1| -----TTTCTAGGACAG-AGGGAG- 18

gb|BG300947.2| TTTTTTTTCTAGGACGGGAGGGAGT 25

- ***CLUSTAL 2.0.12 multiple sequence alignment miR1871***

gb|EX575651.1| TGGCTCTGATATCATGTTG 19

gb|EX594870.1| TGGCTCTGATATCATGTT- 18

gb|EX594871.1| TGGCTCTGATATCATGTT- 18

- ***CLUSTAL 2.0.12 multiple sequence alignment miR2091***

gb|BU999635.1| CAACCGAGCCGAGGAGGA-- 18

gb|BI952042.1| --ACCGAGCCGAGGAGGAGG 18

- ***CLUSTAL 2.0.12 multiple sequence alignment miR2102***

emb|AL504006.1| TGCAGTTGCTGCCTCAAGCTT 21

gb|CX630154.1| -GCAGTTGCTGCCTCAAGCTT 20

gb|BQ754066.1| TGCAGTTGCTGCCTCAAGCTT 21

gb|BQ765438.1| TGCAGTTGCTGCCTCAAGCTT 21

gb|BQ765702.1| TGCAGTTGCTGCCTCAAGCTT 21

gb|CA006693.1| TGCAGTTGCTGCCTCAAGCTT 21

gb|CA008118.1| TGCAGTTGCTGCCTCAAGCTT 21

gb|CA019616.1| TGCAGTTGCTGCCTCAAGCTT 21

gb|CB882722.1| TGCAGTTGCTGCCTCAAGCTT 21

gb|GH228935.1| TGCAGTTGCTGCCTCAAGCTT 21

gb|DN188095.1| TGCAGTTGCTGTCTCAAGCTT 21
