# Supplementary figures and images for: A computational-based update on microRNAs and their targets in barley (Hordeum vulgare L.)
Source: BMC Genomics. 2010 Oct 22;11:595. doi: 10.1186/1471-2164-11-595 (PMC3091740; doi:10.1186/1471-2164-11-595)

Hv.1306


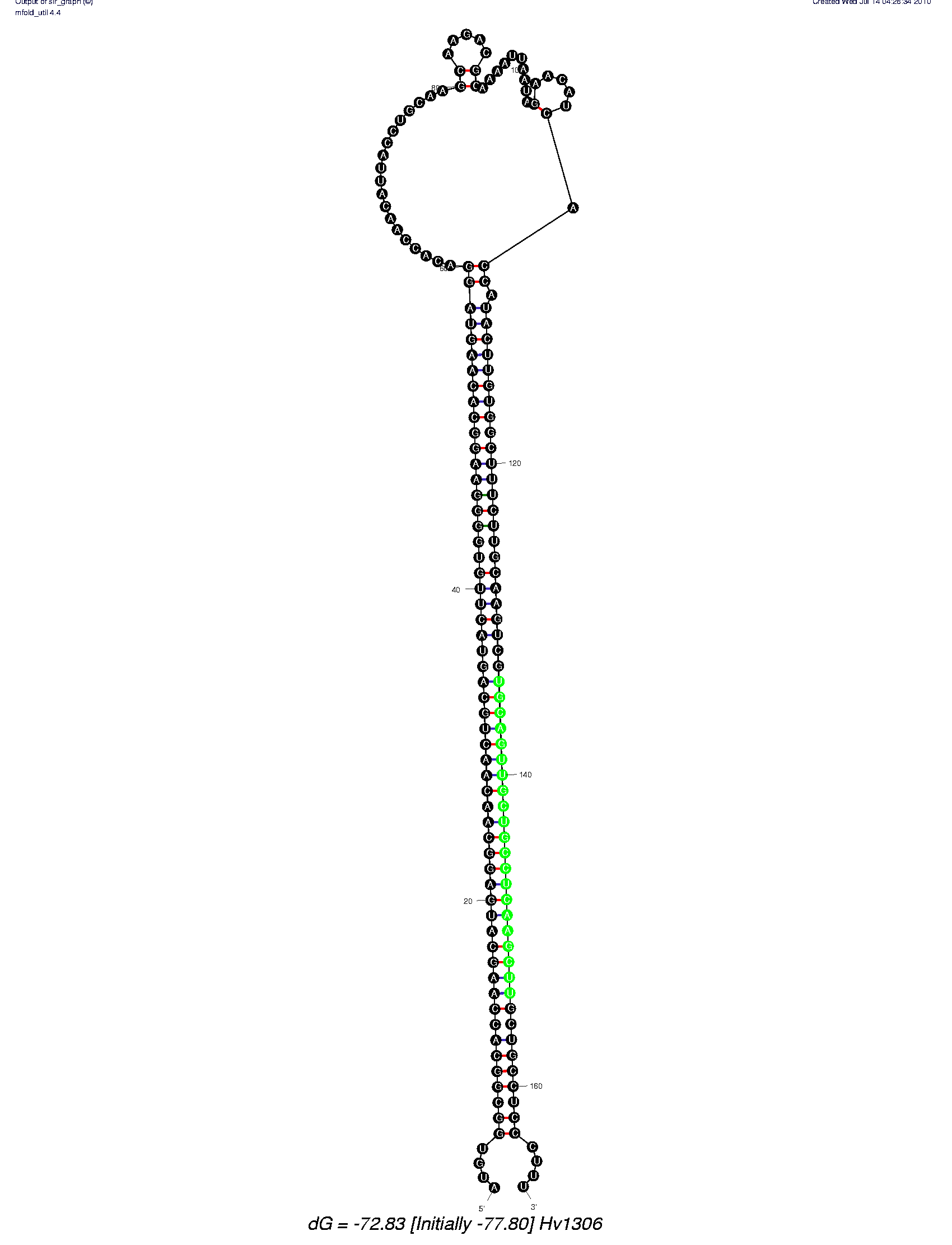


Hv.5064


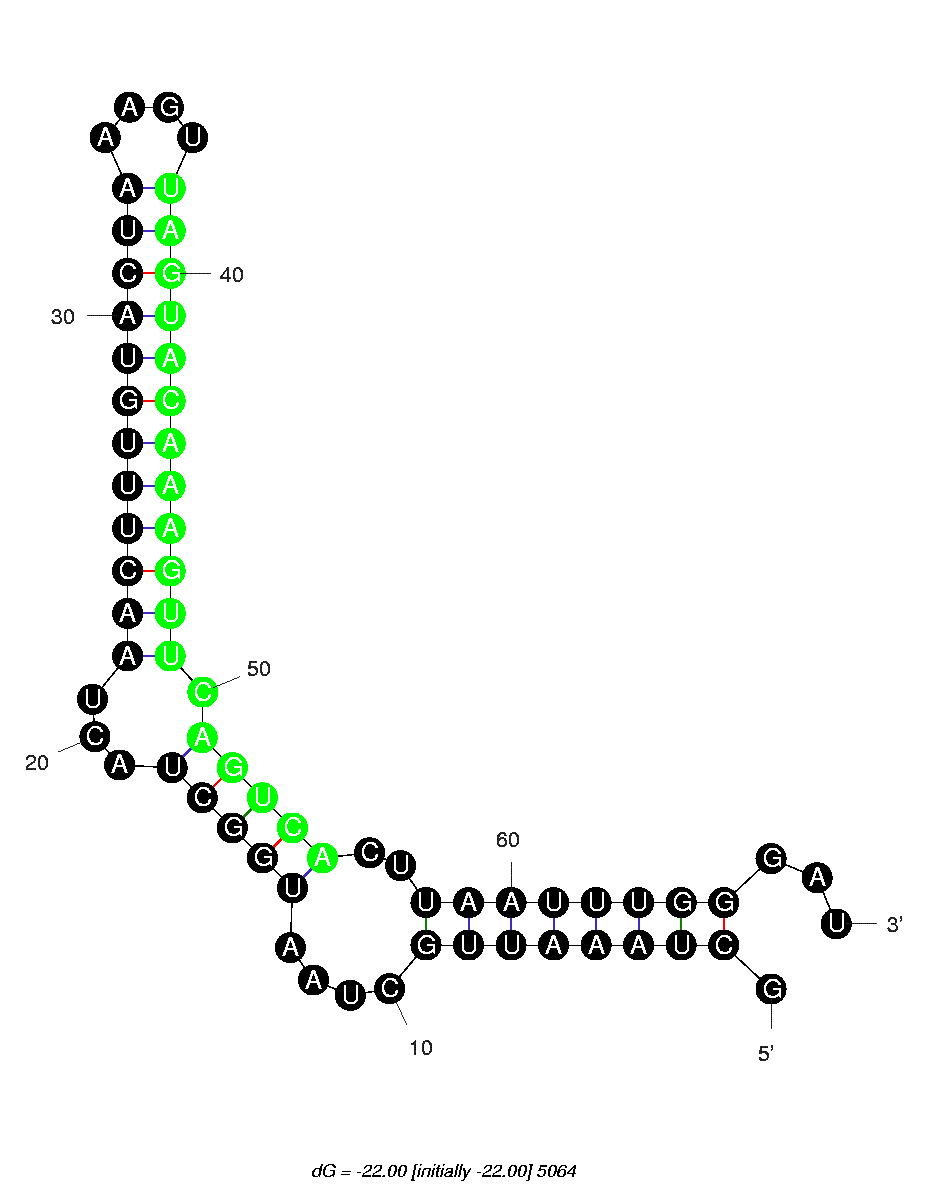


Hv.7117


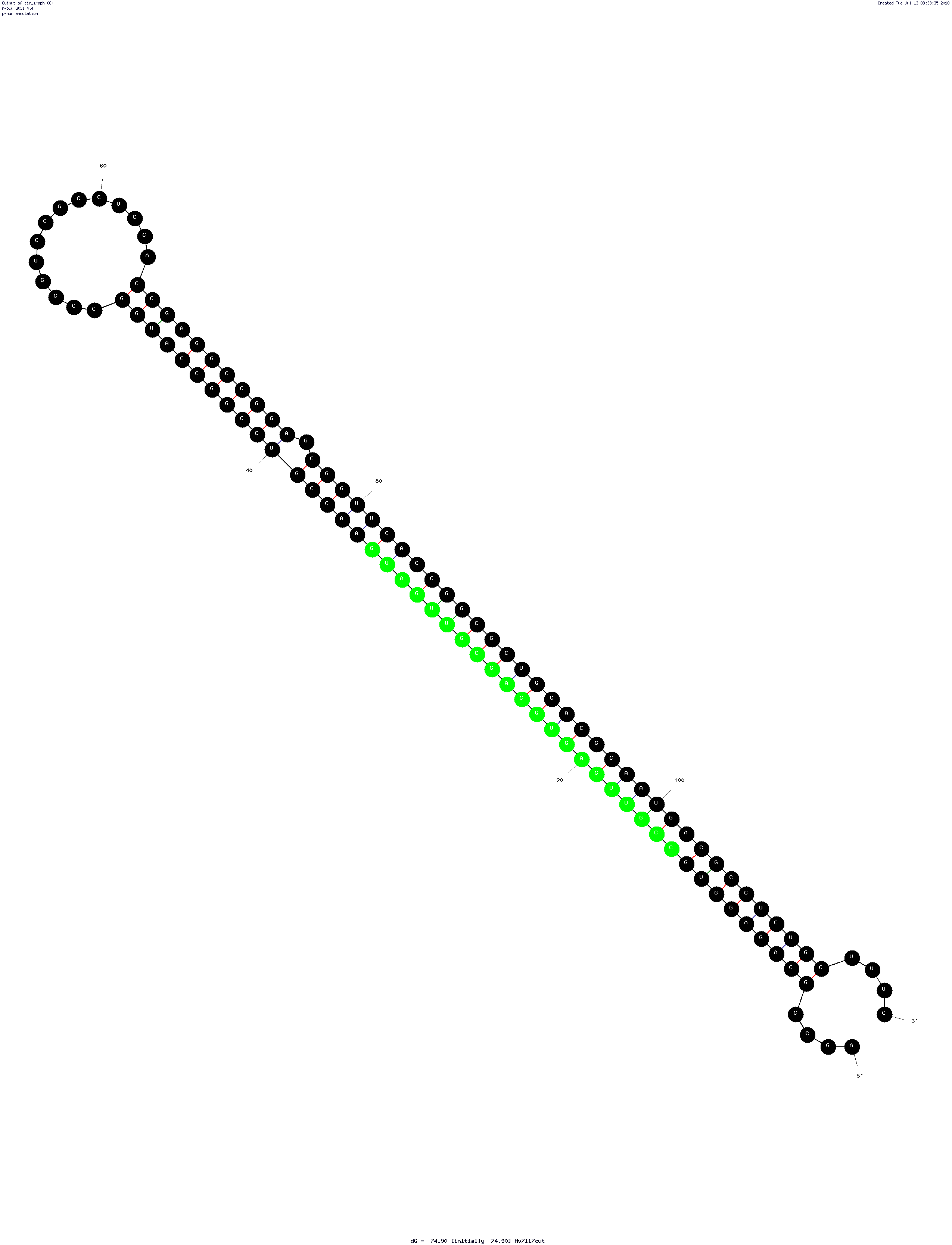


Hv.8158


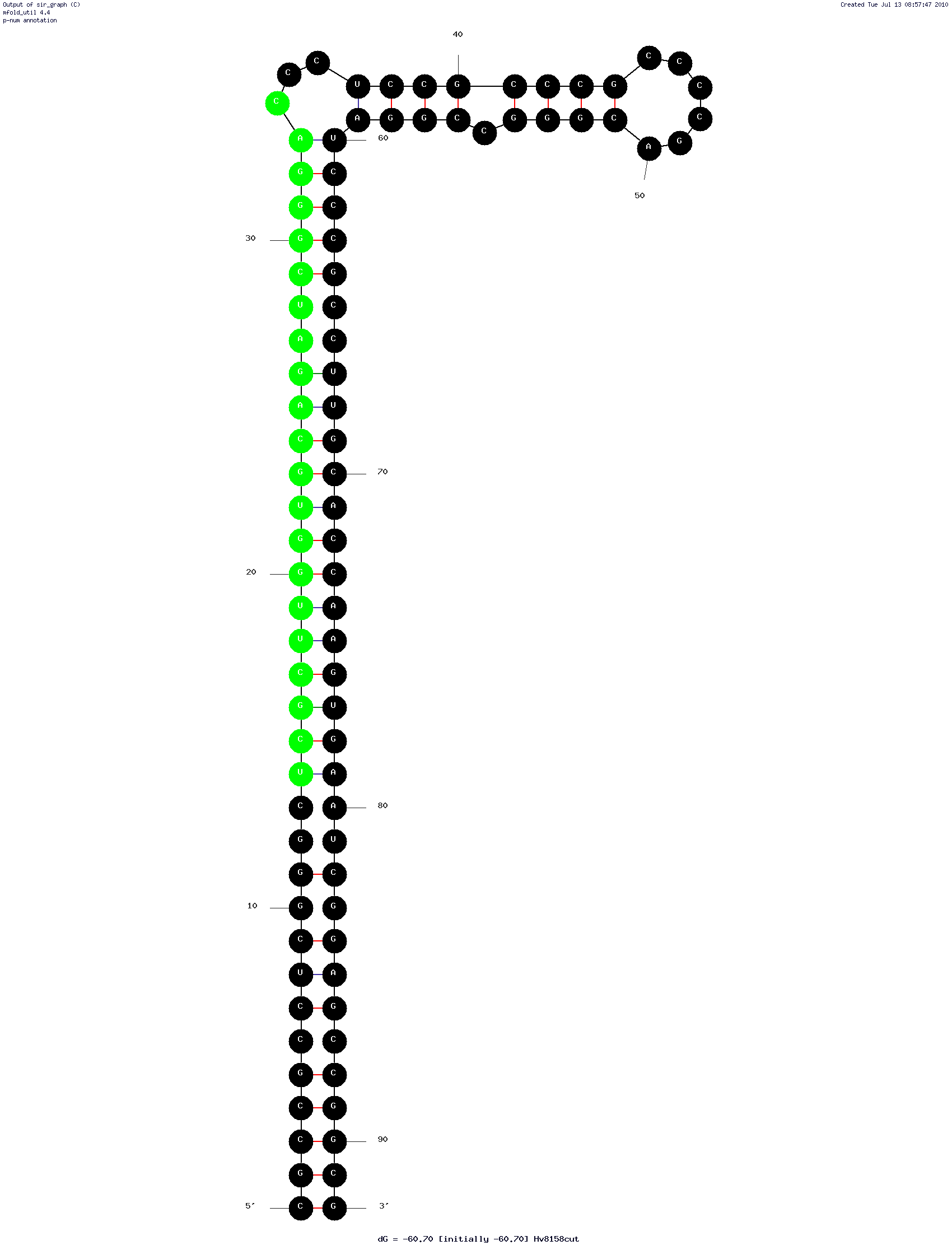


Hv.14657


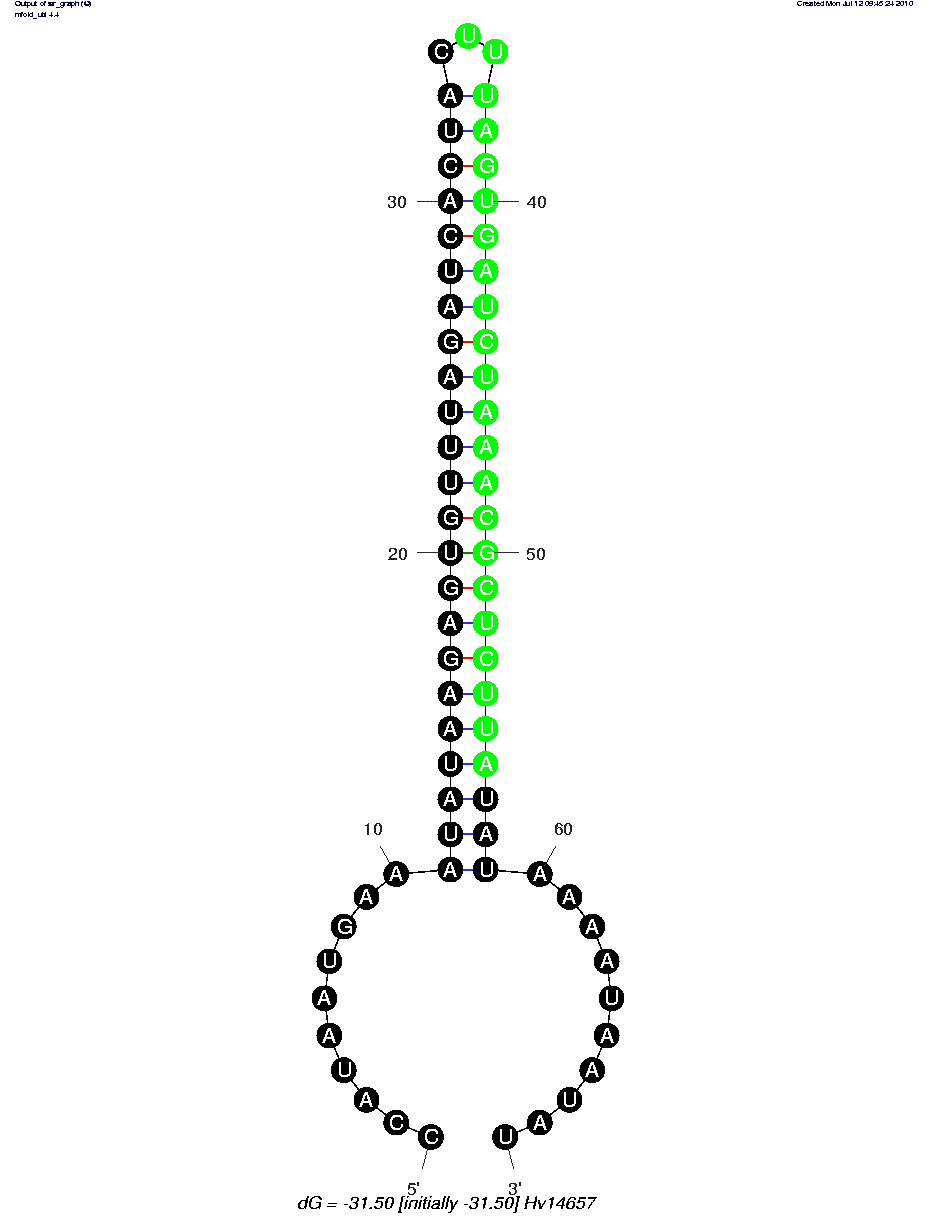


Hv.15131


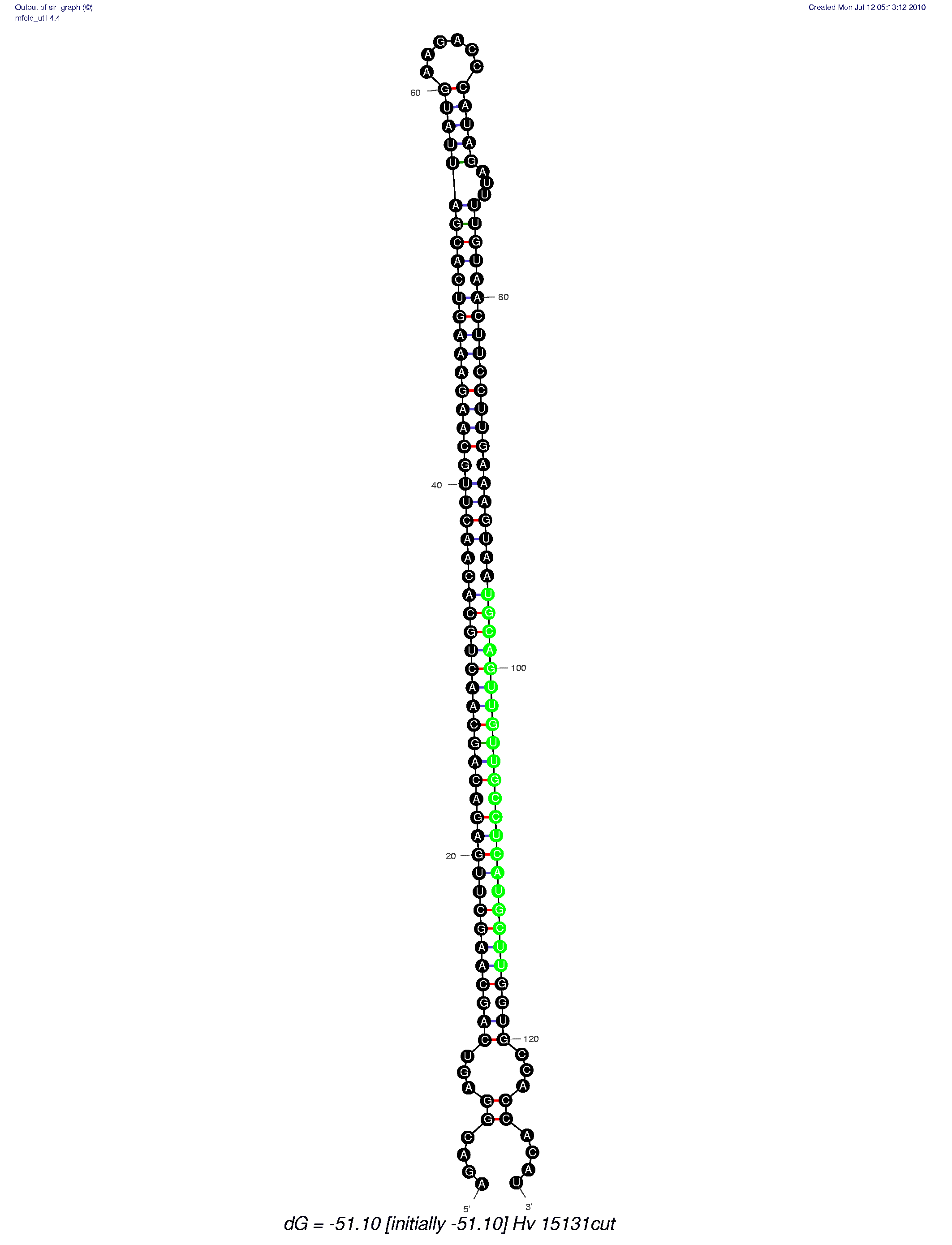


Hv.16635


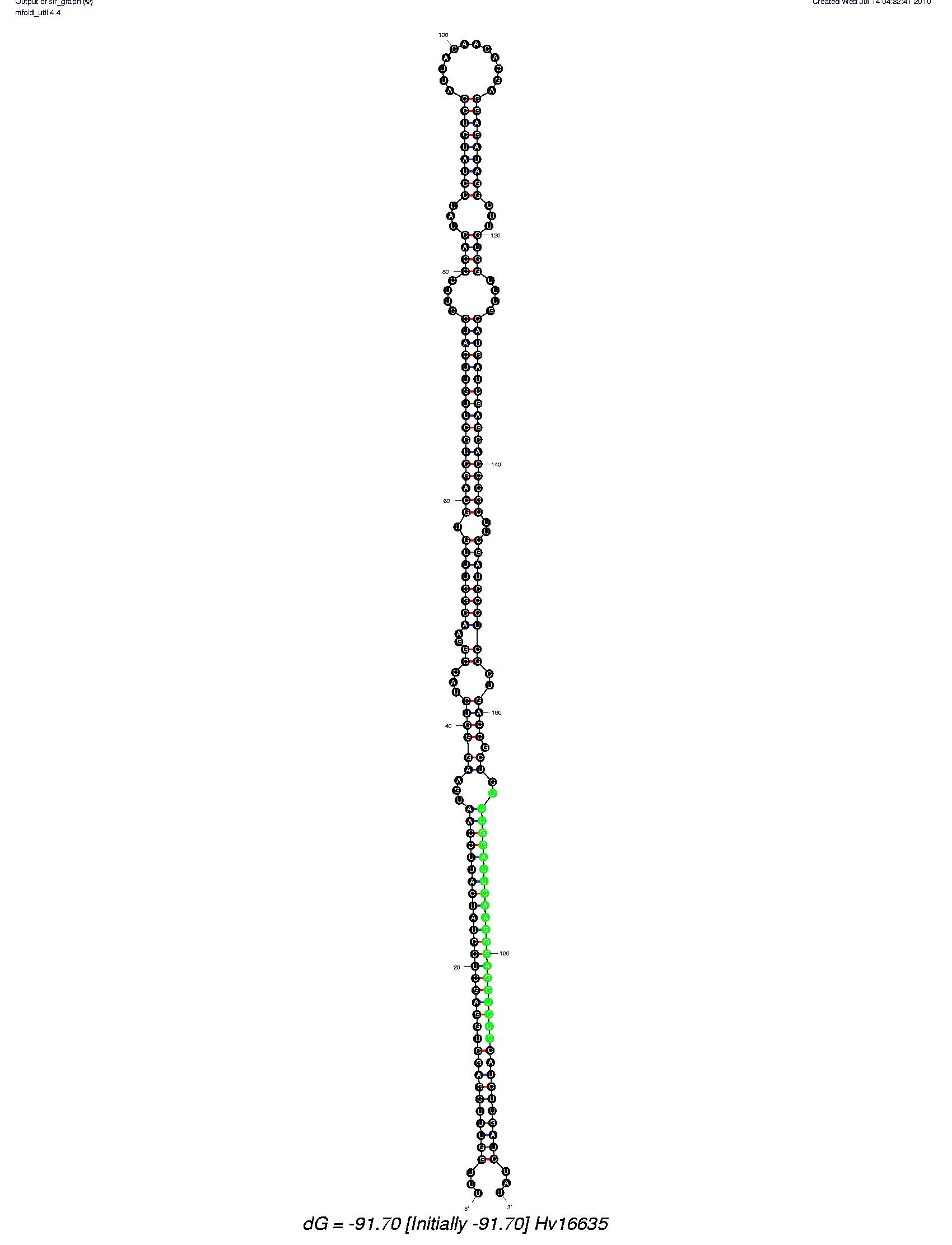


Hv.22601


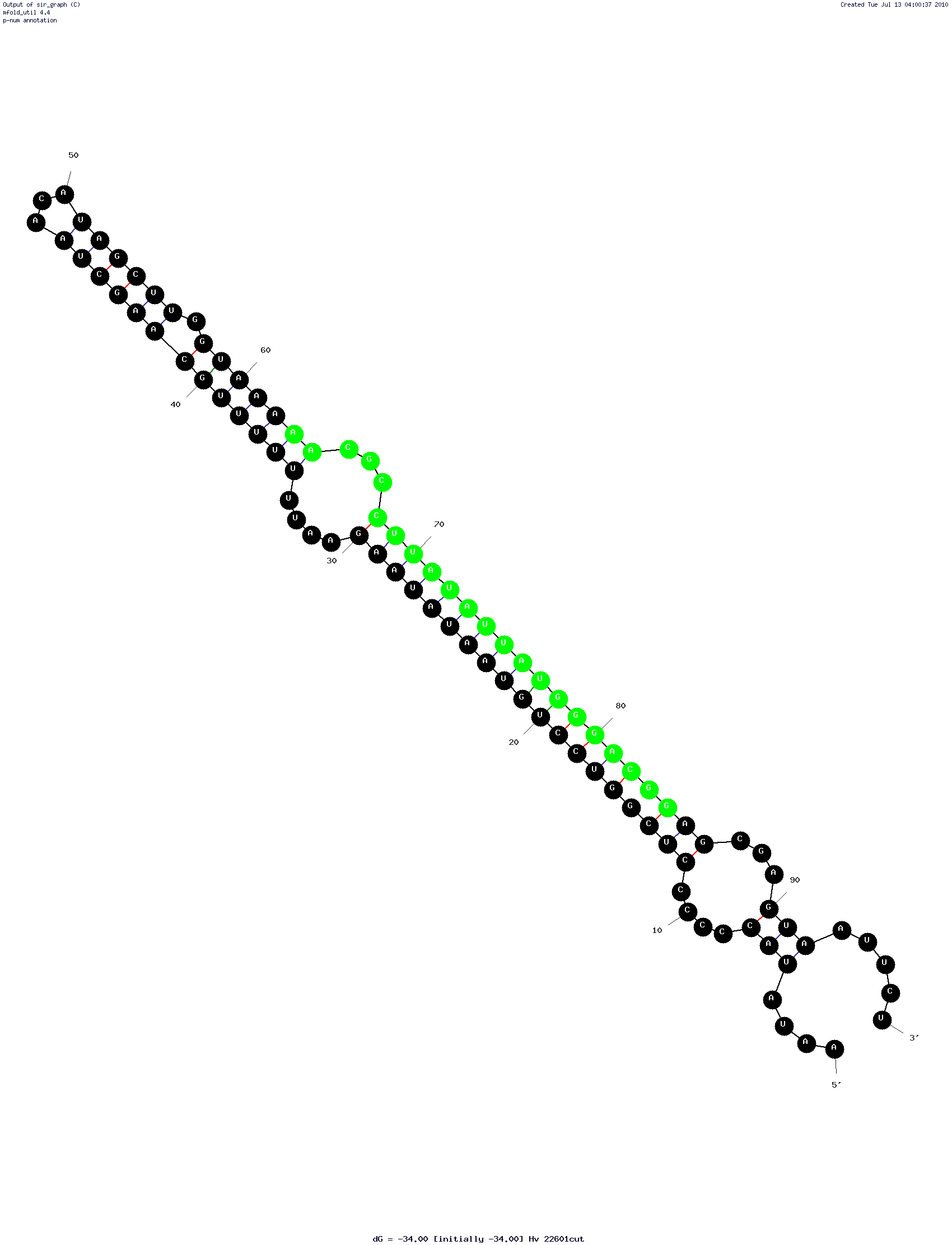


Hv.28058


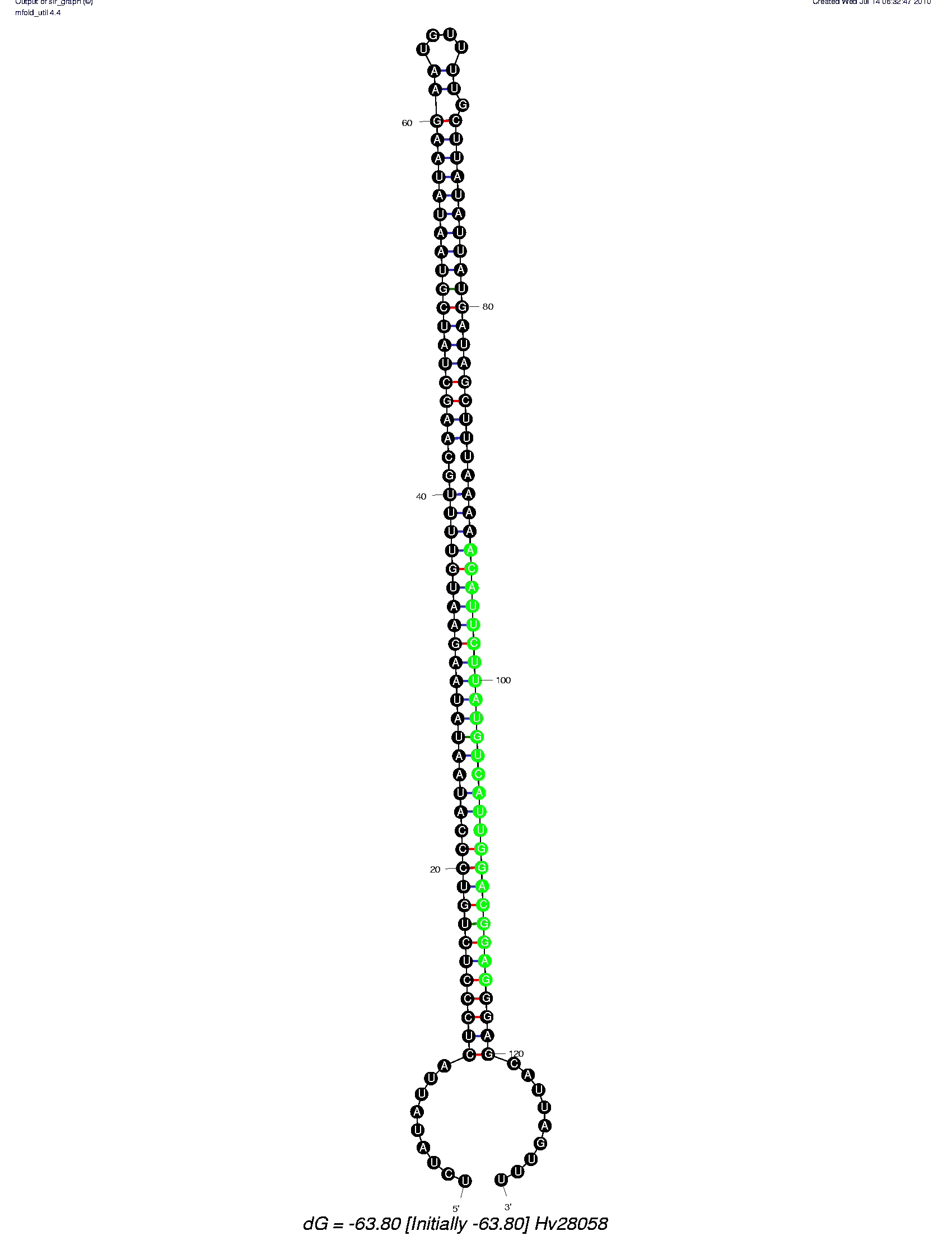


Hv.29065


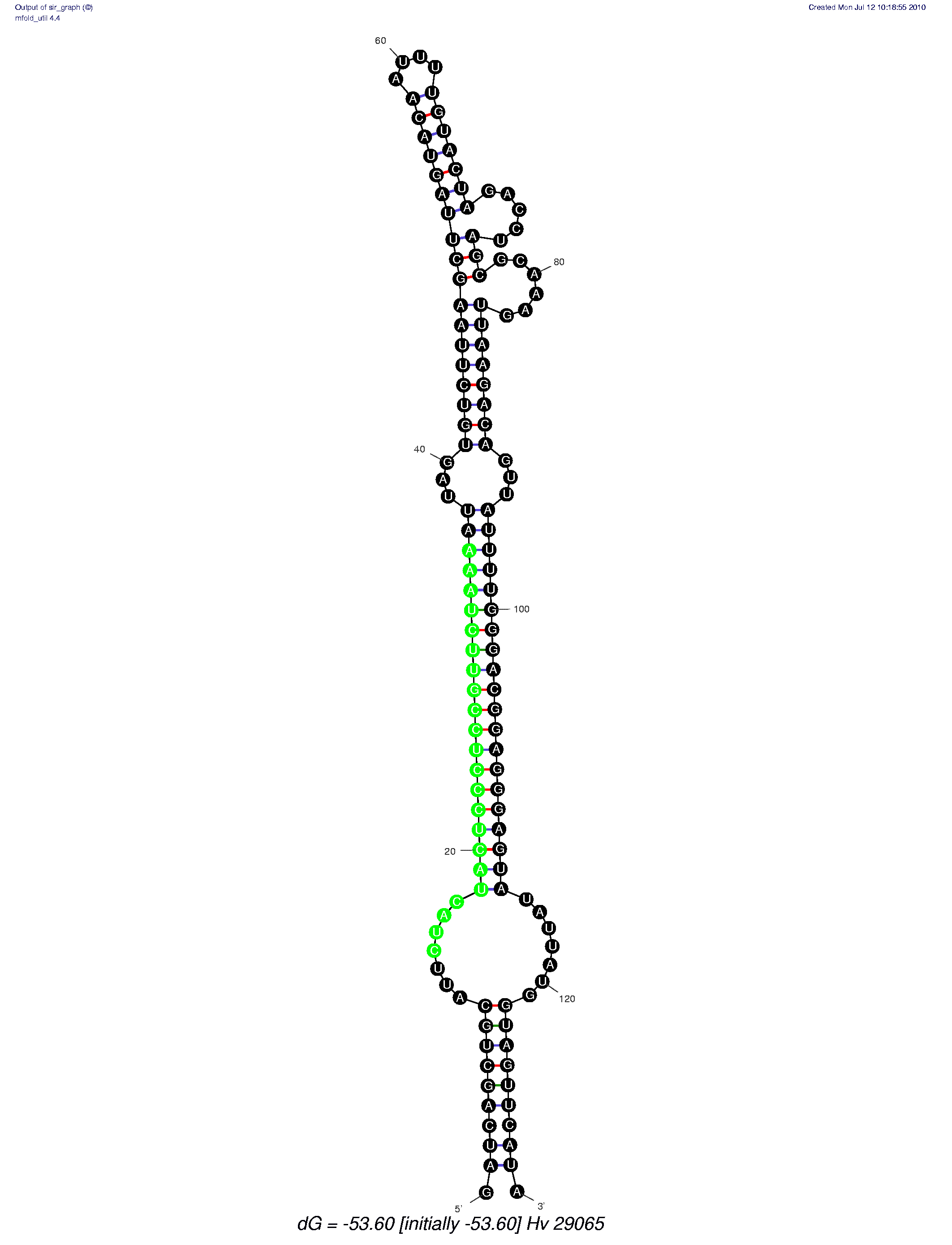


Hv.29519


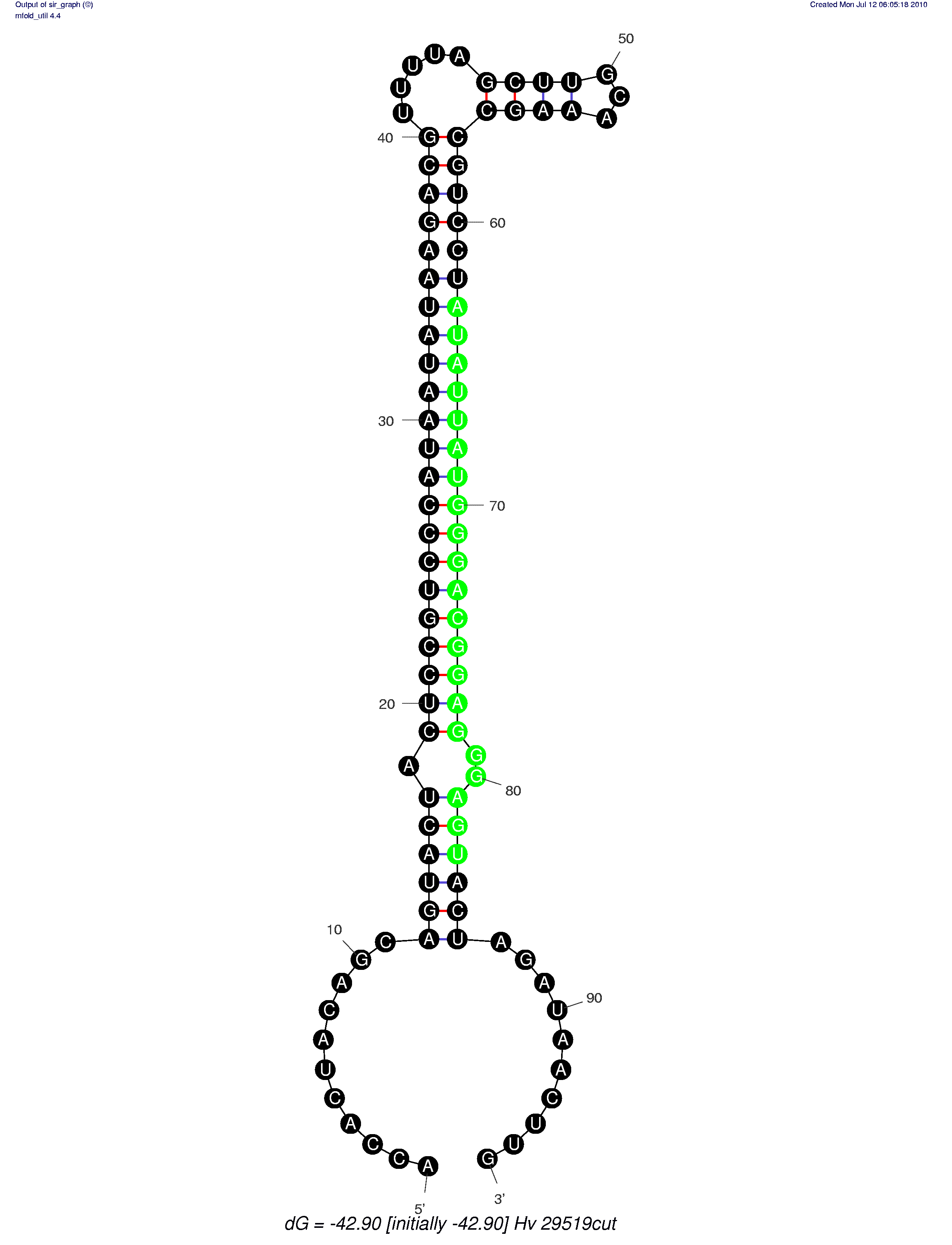


Hv.30469


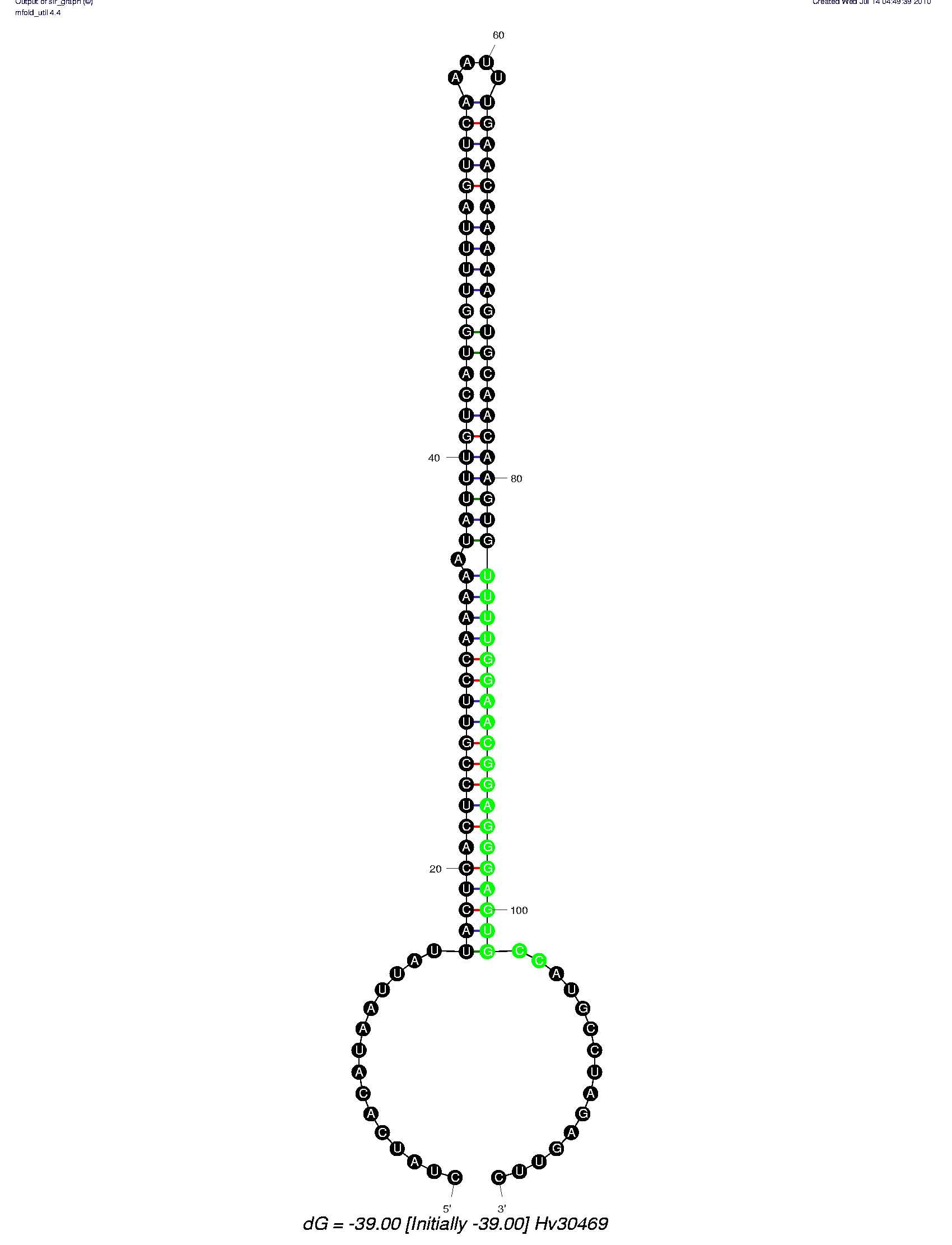

Supplement: Additional file 4 — Secondary structures of the putative miRNA precursors. Predicted structures of the identified putative miRNA precursors. The prediction was performed with mfold. [file 1471-2164-11-595-S4.DOC]
